# Therapeutic Activities and Phytochemical Composition of *Helianthus annuus* L. Extracts

**DOI:** 10.1002/cbdv.202502471

**Published:** 2026-02-16

**Authors:** Marina dos Santos Barreto, Wesley Lisboa de Jesus, Maria Eduarda de Britto Sá, Jessiane Bispo de Souza, Ronaldy Santana Santos, Júlia Santana Lisboa, Pedro Henrique Macedo Moura, Deise Maria Rego Rodrigues Silva, Eloia Emanuelly Dias Silva, Lysandro Pinto Borges, Adriana Gibara Guimarães

**Affiliations:** ^1^ Federal University of Sergipe São Cristóvão Brazil; ^2^ Department of Pharmacy Federal University of Sergipe São Cristóvão Brazil; ^3^ Department of Clinical and Toxicological Analyses School of Pharmaceutical Sciences University of São Paulo (USP) São Paulo Brazil; ^4^ Northeast Biotechnology Network (Renorbio) Federal University of Sergipe São Cristóvão Brazil

**Keywords:** crop residue, *Helianthus annuus*, pharmacology, phytochemistry, sunflower

## Abstract

The sunflower (*Helianthus annuus* L.) is a plant commonly used in agriculture and the fuel industry, as well as being an ornamental garden plant. However, its biologically active compounds make it an interesting plant for medicinal purposes. This review evaluated the phytochemistry of sunflower leaves, stems, receptacles, flowers, seeds, and sprouts and its pharmacological activities. A search was conducted in the PubMed, Embase, ScienceDirect and Google Scholar in March 2025. This review includes studies on the quantitative phytochemical profile of *H. annuus* extract and studies that reported some pharmacological activity of sunflower extracts or metabolites. The compounds identified in the parts of the sunflower include phenolic acids, flavonoids, and terpenes, mainly. These classes of metabolites are responsible for the pharmacological effects of the species, especially 5‐O‐caffeoylquinic acid (chlorogenic acid) and its derivates. Fatty acids, vitamins, alkanes, alkaloids, and benzenoids were also found, but in smaller variations. Studies have reported that the effects of sunflowers include antioxidant, anti‐inflammatory, antimicrobial, antidyslipidemic, hypoglycemic, renal, and colon‐protective activity. Thus, sunflowers are plants rich in chemical compounds with pharmacological potential, which can be used as raw materials for the pharmaceutical industry.

## Graphical abstract

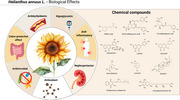

## 1 Introduction

*Helianthus*, a genus belonging to the Asteraceae family, has 67 species [1, 2]. Among these, the most widely propagated species is *Helianthus annuus* L., known as the common sunflower. A native of North America, this species is easy to grow due to the length and depth of its roots, which favor its resistance to changes in temperature and humidity and is currently cultivated on all five continents of the globe [3]. The common sunflower stands out for its biofuel production as a sustainable alternative to the use of diesel [4, 5]. In addition, the dietary oil extracted from sunflower seeds also has important nutritional benefits compared to other oils [6, 7, 8]. This makes the common sunflower the fourth most cultivated oilseed in the world, behind soy, palm, and canola [7].

Sunflower stands out for its antioxidant, anti‐inflammatory, antidiabetic, and antihypertensive properties [6, 9]. This is due to the presence of fiber, proteins, zinc, iron, vitamins, and the metabolite classes flavonoids, phytosides, and phytosterols. Regarding its traditional use, sunflowers have been used to treat coughs, wounds, lung and kidney infections, and fever [10]. The *H. annuus* species has shown promise for human health, as its parts (seeds, stem, leaves, flower) contain phytochemicals of interest for the treatment of various diseases, both chronic and acute. In addition, the phytochemistry and therapeutic value of a species of the same genus as *H. annuus*, *Helianthus tuberosus*, was elucidated in a recent study [11], indicating the therapeutic potential of this genus.

Medicinal plant extracts are a cost‐effective alternative, offering a shorter time to obtain and maintain the active compounds in plants. Extracts have been used for years because they can maintain the phytochemicals present in plants, which are responsible for promoting various pharmacological activities [12]. In addition, extracts provide access to active compounds in plants at a concentration that is not easily accessible [13]. The abundant compounds present in medicinal plants can act synergistically in extracts, taking advantage of various pharmacological activities that would not be possible with the isolated compound, for example [13]. However, it is also possible to identify interesting compounds to be isolated from the extracts.

The widespread use of the seeds in the production of biofuel and oil makes it possible to use the other parts of the plant, such as the leaves, stems, roots, and flowers, which are generally discarded during the industrial process [14]. This makes it an interesting alternative for reducing waste and favoring the economic model [13]. Previous reviews have highlighted the human benefits of *H. annuus* and its mainly metabolities [6, 10, 15]. However, it is still necessary to describe the complete phytochemical profile of all parts of this plant and indicate the reported pharmacological activities. This review aims to compile findings in the literature on the medicinal use of all parts of *H. annuus* in treating conditions affecting human health, focusing on the detailed phytochemistry profile of the whole plant.

## 2 Methodology

### 2.1 Literature Search

A literature search was conducted on the following databases: PubMed, Science Direct, Embase, and Google Scholar. To build the search strategy, the keywords used were “*Helianthus annuus*” OR “sunflower” AND “seeds” OR “flowers” OR “steem” OR “root” AND “phytochemistry” OR “characterization” OR “identification” OR “metabolites” OR “biological activity” and its synonyms. The search was performed properly for each database, using parentheses and/or quotation marks, and the appropriate Boolean operators. The search was conducted in March 2025, and no date limit was added to the search. To be included, the studies had to report the phytochemical profile or some pharmacological activity of the *H. annuus* extract (HAE). Studies that reported therapeutical activities of sunflower metabolites were also included. Studies that only performed a qualitative phytochemical profile of the extract were not included in the review, as well as letters, reviews and conference abstracts. ChemDraw (version 23.1.2 for Windows) was used to represent chemical structures.

### 2.2 Chemical Space Analysis of *H. annuus* Flavonoids

To evaluate the chemical space of the flavonoids identified in *H. annuus*, we carried out an integrative chemoinformatics approach, with the help of Google Colab, focusing on structural characterization and the evaluation of molecular similarity. First, we collected the data of the compounds with confirmed structure (in SMILES format) and standardized names through PubChem. Using the RDKit library, we calculated classical molecular descriptors such as log *P*, molecular weight, number of hydrogen donors and acceptors, among others, which are fundamental for predicting physicochemical properties and pharmacokinetics. To reduce the dimensionality of the data and visualize chemical groupings, we applied principal component analysis (PCA), based on these descriptors. In parallel, we used structural fingerprints of the Morgan type to represent the chemical similarities between the compounds, followed by projection in two dimensions using the t‐SNE algorithm. Both analyses were complemented with classifications according to Lipinski's rules and Veber to assess the oral potential of the compounds. Visualization was carried out with two‐dimensional graphs combining coloring by chemical subclass and differentiation of markers according to compliance with the filters of drug‐likeness (Figure S1).

## 3 Phytochemistry and Nutritional Value of Sunflowers

### 3.1 Nutritional Content of Seeds and Leaves

Guo et al. (2017) highlight the phytochemistry and biological activities of sunflower seeds, pointing to their antioxidant, antimicrobial, antidiabetic, antihypertensive, anti‐inflammatory, and healing effects [6]. These effects may be due to the presence of phenolic compounds (caffeic acid, chlorogenic acid, caffeoylquinic acids, gallic acid, protocatechuic, coumaric, ferulic acid, and sinapic acids), flavonoids (flavanones, flavones, flavonols, isoflavonoids, anthocyanins, chalcone, and aurone), and vitamins A, B, and C [6]. Due to these compounds, the consumption of sunflower seeds leads to important pharmacological activities.

Sunflower seeds stand out for their amount of fatty acids (FAs). The fat content of oil sunflower seeds ranged from 51.9% to 59.4%, and for edible sunflower seeds, from 41.21% to 46.76%, as found by Li et al. [16]. Sunflower seeds and oil are rich in polyunsaturated fatty acid (PUFA) (51.41% and 64.81%, respectively) and monounsaturated fatty acid (MUFA) (41.69% and 20.58%) [8]. The FAs found were mainly oleic (C18:1) and linoleic (C18:2) acids [8, 16, 17]. García‐González et al. evaluated the characterization of oils from pressing and solvent extraction of new sunflower seeds [18]. Similar to what was found in other studies, C18:1 and C18:2 were the dominant (87%–89%) [18]. However, Oliveira et al. (2016) found saturated FAs, predominantly palmitic acid (8.58 g/100 g), followed by stearic acid (3.69 g/100 g) and behenic acid (0.52 g/100 g) [19]. Among the MUFAs, oleic acid (27.27 g/100 g) predominated, followed by palmitoleic acid (0.9 g/100 g). Linoleic acid (LA) (50.68 g/100 g) and α‐linolenic acid (3.37 g/100 g) were the most predominant PUFAs [19]. In addition, Balogun et al. (2023) identified 14 saturated (caproic, caprylic, capric, lauric, myristic, pentacyclic, palmitic, margaric, stearic, arachidic, heneicosyl, behenic, tricosyl, and lignoceric) and 7 unsaturated (oleic, linoleic, paullinic, dihomo‐a‐linoleic, eicosatetraenoic, eicosapentaenoic, nervonic acid) [17]. Ozcan et al. found high amounts of protein and oil content (22.98±0.01 and 41.30 ± 0.50 g/100 g, respectively) in *H. annuus* seeds. Linoleic, oleic, palmitic, and stearic acids were the dominant FAs [20].

Regarding to FAs content in leaves, Fernandez et al. [21] identified in the adult sunflower leaf, linolenic acid, followed by linoleic and palmitic acid, as the most present organic acids. Glucose was the main soluble sugar, and glutamate, alanine and serine were the amino acids most present [21].

These studies show that sunflower seeds are rich in FA content, especially oleic, linoleic, and palmitic acids. LA is an omega‐6 FA important in maintaining skin, hair, metabolism, and immunity. LA is a precursor of proinflammatory eicosanoids, leukotriene, and thromboxanes, which have an important role in regulating immunity. Oleic acid is an omega‐9 FA, and it is responsible for improving lipid profile, maintaining body weight, and preventing neuronal inflammation and insulin resistance. However, these FAs support the body's health and prevent diseases [22].

*H. annuus* seeds also highlight protein content. A study determined the crude protein values of the sunflower ranged from 18.82% to 29.81% [20]. Similarly, the protein content of oil sunflower seeds was 18.46%–22.27%, and for edible sunflower seeds, it was 25.53%–28.53%. The total amino acid content was 39.16 to 47.89 g/100 g, of which the essential amino acid (EAA) content was 13.32–15.87 g/100 g. A higher glutamic acid content was found, followed by aspartic acid and arginine [16]. Petraru et al. (2021) quantified the protein content of sunflower seeds at higher levels (33.85 ± 0.88), and the amino acids most identified in the seeds were aspartic acid, leucine, and glutamic acid [8]. The macroelement most commonly found was calcium, followed by magnesium, potassium, and phosporus [20]. In addition to its elements, 16 were found in seeds (selenium, cesium, calcium, thallium, zinc, magnesium, chromium, copper, nickel, beryllium, cobalt, titan, iron, lithium, molybdenum, and cadmium) [8].

### 3.2 Chemical Compounds

#### 3.2.1 Seeds

Phenolic compounds are abundant in *H. annuus* seeds. Karamać et al. evaluated the total phenolic compounds (TPC) of *H. annuus* kernels [23]. The TPC ranged from 485 ± 5.39 to 18.2 ± 0.62 (mg (+)‐catechin eq/g) in different fractions. 5‐O‐Caffeoylquinic acid (5‐CQA, chlorogenic acid) was the predominant compound in all the extract fractions [23]. Leverrier et al. (2019) also found 5‐CQA as the main compound of the hydroalcoholic extract of sunflower seeds, with CGAs corresponding to 44.6%, and 5‐CQA corresponding to 26.7% w/w [24]. Regarding polyphenolic content, the methanolic extract of the seeds of seven strains of *H. annuus* shows ranged from 22.29 to 33.09 mg gallic acid equivalent (GAE)/g [25]. Chlorogenic acid and caffeic acid were the phenolic compounds found in the greatest quantities [25]. The amount of flavonoids ranged from 1.02 to 0.34 mg rutin equivalents (RE)/g [25]. Phenolic acids and flavonoids were mainly found in the seeds, along with some FAs (Table 1). No studies were found that reported terpenes in the seeds extract, although studies report the terpenoids presence in the seeds [26, 27]. This highlights the importance of phytochemical analyses focused on this therapeutic class.

**TABLE 1:** Chemical compounds, classes, and parts of the sunflower where the compounds were detected.

Number	Compound	Part used	References
Phenolic compounds			
1	(3*R*,4*S*)‐6‐Acetyl‐3,4‐dihydroxy‐2,2‐dimethyl‐chromane	Flowers	[41]
2	1,5‐Di‐*O*‐caffeoylquinic acid (cynarin)	Aerial parts, florets, sprouts	[29, 31, 40]
3	2‐Hydroxy‐5‐acetylbenzoic acid	Flowers	[41]
4	2‐Hydroxycinnamic acid	Seeds	[25, 35]
5	2‐Hydroxymethylimino‐3,4‐dimethyl‐7‐hydroxy‐6‐methyl ketone‐2*H*‐chromon	Flowers	[41]
6	2,5‐Dihydroxybenzoic acid	Seeds	[25]
7	3‐[2‐Formyl‐5‐(hydroxy‐methyl)‐1*H*‐pyrrol‐1‐yl] pentanedioic acid	Flowers	[41]
8	3‐(3‐Methyl‐2‐butenyl)acetophenone‐4‐O‐β‐d‐glucopyranoside	Flowers	[41]
9	3‐O‐Feruloylquinic acid (3‐FQA)	Florets	[29]
10	3‐Hydroxytyrosol	Seeds	[25]
11	3‐Hydroxybenzoic acid	Seeds	[25]
12	3‐O‐(3*S*‐2‐oxo‐3‐hydroxy‐indole‐3‐acetyl)‐5‐O‐caffeoylquinic acid	Seeds	[46]
13	3‐O‐Caffeoylquinic acid	Seeds	[23, 24, 35, 42, 46]
14	3‐O‐*p*‐Coumaroylquinic acid	Seeds	[23]
15	3,4‐Di‐O‐caffeoylquinic acid	Seeds, leaves, aerial parts, florets	[14, 24, 29, 30, 31, 42, 46]
16	3,4‐Dihydroxybenzoic acid	Seeds	[20]
17	3,4‐Dihydroxyphenylacetic acid	Seeds	[25]
18	3,5‐Di‐O‐caffeoylquinic acid (isochlorogenic acid)	Seeds, leaves, aerial parts, florets	[14, 24, 29, 30, 31, 35, 42, 46]
19	4‐Hydroxy‐3‐((*Z*)‐3′‐hydroxy‐3′‐methyl‐1′‐butenyl) acetophenone‐6‐O‐β‐d‐glucopyranoside	Flowers	[41]
20	4‐Hydroxy‐3‐((*Z*)‐3′‐hydroxy‐3′‐methyl‐1′‐butenyl) acetophenone‐8‐O‐β‐d‐glucopyranoside	Flowers	[41]
21	4‐Hydroxy‐3‐(2′‐hydroxy‐3′‐methyl‐1′‐butenyl) acetophenone‐1′‐O‐β‐d‐glucopyranoside	Flowers	[41]
22	4‐Hydroxybenzoic acid	Seeds	[25, 37]
23	4‐O‐(3*S*‐2‐oxo‐3‐hydroxy‐indole‐3‐acetyl)‐5‐O‐caffeoylquinic acid	Seeds	[46]
24	4‐O‐Caffeoylquinic acid (4‐CQA)	Seeds	[23, 24, 35, 42, 46]
25	4‐O‐Feruloylquinic acid (4‐FQA)	Florets	[29]
26	4‐O‐*p*‐Coumaroylquinic acid	Seeds	[23]
27	4,5‐di‐O‐caffeoylquinic acid	Seeds, leaves, aerial parts, florets	[14, 24, 29, 30, 31, 42, 46]
28	4,6‐Hydroxy‐3‐((*Z*)‐3′‐hydroxy‐3′‐methyl‐1′‐butenyl) acetophenone‐8‐O‐β‐d‐glucopyranoside	Flowers	[41]
29	5‐O‐Caffeoylquinic acid (5‐CQA) (chlorogenic acid)	Seeds	[14, 23, 24, 25, 29, 30, 31, 35, 37, 42, 46]
30	5‐O‐Feruloylquinic acid (5‐FQA)	Florets, seeds	[29, 42]
31	5‐O‐*p*‐Coumaroylquinic acid	Seeds	[23, 42]
32	6‐Acetyl‐2,2‐dimethylchromene‐8‐O‐β‐d‐glucoside	Flowers	[41]
33	β‐d‐Apiofranosyl‐(1→6)‐β‐d‐(4‐O‐caffeoyl) glucopyranoside	Seeds	[35]
34	Caffeic acid	Seeds and sprouts	[20, 25, 35, 36, 37, 42, 46]
35	Caffeic acid hexose	Aerial parts, florets	[29, 30, 31]
36	Caffeoyl‐dimethoxycinnamoylquinic acid (isomers 1–5)	Seeds	[23]
37	Caffeoylferuloylquinic acid	Seeds, florets	[23, 29]
38	Cinnamic acid	Seeds	[20, 37]
39	*cis*‐Ferulic acid	Seeds	[23]
40	Cryptochlorogenic acid	Aerial parts, florets	[30]
41	Crotonine	Flowers	[41]
42	Dicaffeoylquinic acids (isomers 1–6)	Seeds	[23]
43	Dihydroretinoic acid	Petals	[33]
44	Erigeside II	Flowers	[41]
45	Eriodicthiol 5‐O‐β‐d‐glucoside	Seeds	[35, 46]
46	Ferulic acid	Seeds and sprouts	[20, 25, 36, 37, 42]
47	Ferulic acid dehydrotrimers 1–6	Seeds	[23]
48	Gallic acid	Seeds and sprouts	[20, 25, 36, 37]
49	Gentisic acid	Seeds	[37]
50	Homovanillic acid	Seeds	[25]
51	Hydroxytyrosol	Seeds	[37]
52	Isoferulic acid	Florets	[29]
53	Lukianol A	Petals	[33]
54	Methyl‐5‐hydroxy‐2‐pyridinecarboxylate	Flowers	[41]
55	Methyl caffeoate	Seeds	[35]
56	Mehtyl chlorogenate	Seeds	[35]
57	Neochlorogenic acid	Aerial parts	[30]
58	*o‐*Coumaric acid	Seeds	[37]
59	*p*‐Coumaric acid	Seeds and sprouts, florets	[20, 25, 36, 37]
60	*p*‐Coumaric acid hexose	Florets	[29, 31]
61	Pinoresinol	Seeds, leaves	[25, 43]
62	Protocatechuic acid	Seeds and sprouts	[25, 36, 37]
63	Pyrocatechol	Seeds	[25]
64	Ramalinoic acid (benzoic acid, 2,4‐dihydroxy‐3‐[(2‐hydroxy‐4‐methoxy‐6‐propylbenzoyl) oxy]‐6‐pentyl‐)	Petals	[33]
65	Rosmarinic acid	Seeds	[25]
66	Salicylic acid	Seeds	[37]
67	Sinapic acid	Seeds and sprouts	[25, 36]
68	Syringic acid	Seeds	[20, 25, 37]
69	*trans*‐Ferulic acid	Seeds	[23]
70	*trans*‐*m*‐Hydroxycinnamic acid	Seeds	[37]
71	Vanillic acid	Seeds	[25, 37]
72	Vanillin	Seeds	[25]
Flavonoids			
73	(−)‐Epicatechin	Seeds	[25]
74	(2*S*,3*S*)‐3,5,7‐trihydroxy‐2‐(4‐hydroxyphenyl)‐8‐(3‐methyl‐2‐buten‐1‐yl)‐2,3‐dihydro‐4*H*‐chromen‐4‐one	Receptacles	[38]
75	1,5‐Anhydro‐6‐deoxy‐2‐O‐(6‐deoxyl‐a‐l‐mannopyranosyl)‐1‐[5,7‐dihydroxy‐2‐(4‐hydroxyphenyl)‐4‐oxo‐4*H*‐chromen‐6‐yl]hexitol	Receptacles	[38]
76	3‐(3‐Methyl‐2‐butenyl) acetophenone‐4‐O‐β‐d‐glucopyranoside	Flower	[41]
77	3,5,7‐tri‐O‐methyl‐4′‐O‐(trimethylsilyl) kaempferol	Petals	[33]
78	5‐O‐Methylgenistein	Receptacles	[38]
79	5,2′‐Hihydroxy‐6,7,8,6′‐tetramethoxylflave	Receptacles	[38]
80	Aesculetin	Flowers	[41]
81	Annuolide A‐15‐O‐β‐d‐glucopyranoside	Flowers	[41]
82	Apigenin	Seeds and petals	[36]
83	Apigenin 5‐O‐glucuronide	Petals	[33]
84	Apigenin 6‐C‐arabinosyl‐8‐C‐glucoside	Petals	[33]
85	Apigenin 7‐glucoside	Seeds	[25]
86	Catechin	Seeds	[20]
87	Cirsiliol	Receptacles	[38]
88	Daidzein	Receptacles	[38]
89	EGCG‐5′‐O‐α‐glucopyranoside (Inoveol EGCG)	Petals	[33]
90	Eriodictyol	Seeds	[37]
91	Flavanone	Seeds	[23]
92	Heliannone A	Petals	[33]
93	Heliannone B	Petals	[33]
94	Heliannuoside A	Flowers	[41]
95	Hesperidin (β‐7‐rutinoside of hesperetin)	Seeds	[25]
96	Hispidulin	Receptacles	[38]
97	Hyperoside (quercetin‐3‐O‐galactoside)	Seeds	[25]
98	Isoquercetin	Receptacles	[38]
99	Isoquercitrin	Aerial parts, florets	[29, 31]
100	Jaceosidin	Receptacles	[38]
101	Kaempferol	Seeds	[20, 36]
102	Narwogonin 5,7,8 trihydroxyflavone 7‐glucuronide	Petals	[33]
103	Luteolin	Petals, seeds	[25, 37]
104	Luteolin 7‐O‐diglucuronide	Petals	[33]
105	Luteolin 7‐glucoside	Petals	[33]
106	Naringenin‐7‐O‐glucuronide	Petals	[33]
107	Pectolinarigenin	Receptacles	[38]
108	Pelargonidin‐3‐O‐glucoside	Petals	[33]
109	Quercetin	Seeds	[20, 36]
110	Quercetin 3,5‐diglucoside	Petals	[33]
111	Quercetin derivative	Seeds	[23]
112	Quercetin diglycoside	Seeds	[23]
113	Quercetin glucuronide	Seeds	[23]
114	Quercetin rutinoside	Seeds	[23]
115	Resveratrol	Seeds	[20]
116	Rutin	Seeds	[20, 37]
117	Scrophulein	Receptacles	[38]
118	Silibinin	Receptacles	[38]
119	Tambulin	Leaves	[43]
120	Tricin 5‐O‐β‐d‐glucoside	Receptacles	[38]
Terpenoids			
121	(−)‐kaur‐16‐en‐19‐oic acid (kaurenoic acid)	Leaves, flowers, heads	[28, 41, 43, 45]
122	(+)‐pinocarveol 3‐O‐β‐d‐glucopyranoside	Flowers	[41]
123	(−)‐pinocarveol 3‐O‐β‐d‐glucopyranoside	Flowers	[41]
124	(4*R*, 6*S*)‐carveol β‐d‐glucoside	Flowers	[41]
125	(*E*,*E*)‐4,8,12‐Trimethyl‐1,3,7,11‐tridecatetraene	Petals	[33]
126	(*S*)‐*cis*‐verbenol	Petals	[33]
127	1,1‐Dicyclopentylethane	Petals	[33]
128	l‐Terpinen‐4‐ol	Petals	[33]
129	1,6‐Dimethyl‐9‐(1‐methylethylidene)	Petals	[33]
130	1(7),5, 8‐o‐Menthatriene	Petals	[33]
131	1‐Oxo‐3‐α‐(4‐methyl‐3‐pentenyl)‐6‐methyl‐1,3,3	Petals	[33]
132	10‐oxo‐isodauc‐3‐en‐15‐al	Leaves	[43]
133	10β,14‐dihydroxy‐11α*H*‐guaia‐4 (15)‐ene‐12,6α‐olide	Flowers	[41]
134	15‐α‐angeloyloxy‐ent‐kaur‐16‐en‐19‐oic acid	Leaves	[28]
135	17‐hydroxy‐16 α‐kauran‐19‐oic acid	Leaves	[43]
136	1β,11‐dihydroxy‐5‐eudesmene	Flowers	[41]
137	2,4‐di(trimethylsiloxy)‐6,7‐(methylenedioxy)‐2*H*‐1,4‐benzoxazin‐3‐one	Petals	[33]
138	3‐(Dodecenyl) dihydro‐2,5‐furandione	Petals	[33]
139	3,6‐hydroxy‐5,6‐dihydro‐β‐ionol	Leaves	[43]
140	4‐Bromo‐4‐cholesten‐3‐one	Petals	[33]
141	4‐Chloro‐1‐(4‐chlorophenylsulfonyl)benzene	Petals	[33]
142	Angelic acid	Leaves	[28]
143	Aromadendrene	Receptacles, petals	[33]
144	β‐Amyrin	Leaves, petals	[33, 43]
145	β‐Sitosterol trimethylsilyl ether	Petals	[33]
146	Bicyclo [3.1.1] hept‐3‐en‐2‐one, 4,6,6‐trimethyl Levoverbenone	Petals	[33]
147	Calarene	Petals	[33]
148	Caryophyllene oxide	Petals	[33]
149	Cinnamaldehyde	Petals	[33]
150	Cycloisolongifolene	Petals	[33]
151	Eupatoriochromene	Petals	[33]
152	Germacrene D	Petals	[33]
153	Grandifloric acid	Leaves and heads	[43, 45]
154	Heliannuol A	Leaves	[43]
155	Heliannuol C	Leaves	[43]
156	Heliannuol D	Leaves	[43]
157	Helianthoside 1	Petals	[44]
158	Helianthoside 2	Petals	[44]
159	Helianthoside 3	Petals	[44]
160	Helianthoside 4	Petals	[44]
161	Helianthoside 5	Petals	[44]
162	Helianthoside B	Petals	[44]
163	Lanosterol	Petals	[33]
164	Sclarene	Petals	[33]
165	Stigmasterol	Leaves	[33, 43]
166	Thymol‐β‐d‐glucopyranoside	Petals	[33]
167	Trachylobanoic acid	Head Flower	[45]
168	*trans*‐(+)‐Carveol	Petals	[33]
169	*trans*‐Caryophyllene Bicyclo [7.2.0] undec‐4‐ene	Petals	[33]
170	*trans*‐Verbenol	Petals	[33]
171	Verbenyl ethyl ether	Petals	[33]
172	Zingiberene	Petals	[33]
173	α‐Selinene	Petals	[33]
174	α‐Amyrin	Leaves	[43]
175	α‐Fenchyl acetate bicyclo [2.2.1] heptan‐2‐ol	Petals	[33]
176	α‐Humulene	Petals	[33]
177	α‐Ylangene	Petals	[33]
178	β‐Elemene cyclohexane	Petals	[33]
179	β‐Selinene naphthalene, decahydro‐4a‐methyl	Petals	[33]
180	δ‐Cadinene	Petals	[33]
Fatty acids			
181	1,2‐Diacylglycerol	Pollen and stigma	[39]
182	1,3‐Diacylglycerol	Pollen and stigma	[39]
183	2‐Linoleoyl glycerol	Seeds, leaves	[43]
184	Arachidic acid	Seeds, florets	[20, 29]
185	Behenic acid	Seeds, florets	[20, 29]
186	*cis*‐11‐Eicosenoic acid	Pollen and stigma	[3]
187	Hexadecanoic acid	Petals	[33]
188	Lauric acid	Seeds, florets	[29]
189	Lignoceric acid	Seeds, pollen, florets	[29, 39]
190	Linoleic acid	Seeds, pollen and stigma, florets	[20, 29, 39]
191	Linolenic acid	Pollen and stigma, florets	[20, 29, 39]
192	Margaric acid	Florets	[29]
193	Myristic acid	Seeds, florets	[20, 29]
194	Oleic acid	Seeds, pollen and stigma, florets	[20, 29, 39]
195	Palmitic acid	Seeds, pollen and stigma, florets, petals	[20, 29, 39]
196	Stearic acid	Seeds, pollen and stigma, florets	[20, 29, 39]
Vitamins			
197	Vitamin B_1_	Florets	[29]
198	Vitamin B_2_	Florets	[29]
199	Vitamin C	Florets	[29]
200	Vitamin E	Florets, petals	[29, 33]
Alkanes			
201	Hexadecane	Petals	[33]
202	Octadecane	Petals	[33]
203	Pentadecane	Petals	[33]
Alkaloids			
204	2,3‐Dedimethylcolchicine	Petals	[33]
205	3‐(2‐Phenylethyl)‐1,2,4,4a,5,6‐hexahydropyrazino [1,2‐*a*]quinoline, hydrochloride	Petals	[33]
206	4‐[2‐Formyl‐5‐(methoxymethyl)‐1*H*‐pyrrol‐1‐yl] butanoic acid	Flowers	[41]
207	4‐[Formyl‐5‐(methoxymethyl)‐1*H*‐pyrrol‐1‐yl] butanoate	Flowers	[41]
208	3,3′:5,3″‐bis (trimethylene)‐2,6‐di(1′,8′‐naphthyrid‐2′‐yl)pyridine	Petals	[33]
Benzenoids			
209	1,2‐Benzenedicarboxylic acid, dibutyl ester	Flowers	[33]
210	Benzoic acid, 2,4‐bis[(trimethylsilyl)oxy]‐, trimethylsilyl ester	Flowers	[33]
211	Phthalic acid diethyl ester	Flowers	[33]

#### 3.2.2 Leaves

Although they are not as widely propagated as the seeds, the *H. annuus* leaves have compounds that promote important pharmacological effects. These effects may be due to its composition rich in phenolic compounds, terpenes, and others. A study by Fernandez et al. [21] analyzed the sunflower leaf extract via LC–ESI–QTOF–MS, generating two intense peaks of mono‐ and dicaffeoylquinic acids of the caffeoylquinate class. Methylflavonoids and sesquiterpenes were also identified in the *H. annuus* leaves [21]. Móricz et al. suggested the identification of caurenoic acid ditrpenic acid, anngelic acid, and two sesquisterpenes, possibly helianuols [28]. Derivatives of chlorogenic acid, tambulin as a flavonoid, and 13 different terpenes were found in the leaves (Table 1).

#### 3.2.3 Stems and Flowers

Although they are generally discarded or used for other than medicinal purposes, the stems and flowers of *H. annuus* have been studied for their rich variety of bioactive compounds. Liang et al. (2013) evaluated the ray florets (Rf) and disc florets (Df) of *H. annuus* as a source of fiber [29]. The total dietary fiber in Rf and Df was determined as 42.90 and 58.97 g/100 g, respectively. Potassium was identified as the most abundant mineral element in both Rf and Df, with levels of over 1900 mg/100 g [29]. Palmitic acid was the most abundant saturated FA and linoleic, linolenic and oleic acids were the unsaturated FAs most abundant [29]. These three acids make up more than 90% of the unsaturated FAs in Rf and Df. The total amounts of EAAs in Rf and Df were 3.21 and 3.09 g/100 g, respectively. The most abundant EAAs were valine (0.67 g/100 g) and leucine (0.65 g/100 g) in Rf and Df, respectively [29].

The study by Gai et al. (2020) evaluated the phenolic profile of *H. annuus* from the aerial parts of sunflowers harvested at five growth stages, from stem extension to late flowering. The phenolic compounds ranged from 17.6 to 29.3 mg GAE/g, highlighting the middle flowering stage of the plant [30]. Ye et al. (2015) evaluated the effects of solvents on the composition and phenolic content of *H. annuus* florets [31]. The 90% (v/v) methanol extract led to the highest amount of phenolic compounds (2787.2 mg GAE/100 g) [27]. The study by Li et al. evaluated the composition of *H. annuus* flower extract. Sunflower flower powder had higher concentrations of protein (22.16 ± 2.32%), polysaccharide (27.54 ± 1.64%), reducing sugars (16.39 ± 1.42%), flavonoids (14.61 ± 0.31% × 10), alkaloids (11.76% × 10), and triterpenes (6.08 ± 0.22) [32].

In 2025, Abbaschian and Soltani evaluated the sunflower petal extract phenolic compound by GC–MS [33]. The concentration of ethanol had a significant effect on the total content of phenolic compounds, with the 99.9% ethanol extract being most effective in extraction (63.33 ± 0.00 g GAE/L of extract). The predominant volatile compound in the sunflower extract (SFE) was germacrene D (18.01 ± 0.68%). The most abundant phenolic compound in SFE is heliannone A (25.14 ± 0.02%) [33]. In other study, the levels of free phenolics detected in Rf and Df were 1685 and 2513 mg/100 g, respectively, 89.69% in Rf and 88.71% in Df. 1,5‐diCQA was identified as the most abundant free phenolic compound in both Rf and Df, followed by isoquercitrin in Rf and 4,5‐diCQA in Df [29].

Terpenes and phenolic compounds were found mainly in the aerial parts of the plant, such as the stem and flowers. Terpenes (49 compounds) and phenolic compounds were found in greater diversity (30 compounds) in the aerial parts as a whole. Terpenes, flavonoids, as well as benzenoids and alkaloids, were found in the flowers, florets and petals. Thirteen flavonoids and one terpene were found in the receptacles (Table 1).

#### 3.2.4 Husks

Li et al. evaluated the potential of sunflower husk extract and found a polyphenol content (g/dry biomass) of 6.46 mg of ascorbic acid equivalents (AsA) and 29.68 mg of chlorogenic acid equivalents (CHA) [34]. The flavonoid content was 9.70 µg of quercetin equivalents (QE) and 15.93 µg of RE. The sunflower husk extract stood out for the presence of phenylpropanoids and polyketides, organic acids, and benzenoids, especially polyphenols, which stand out for their antioxidant activity. In addition to tannins, flavonoids, coumarins, and isoflavonoids, eleven classes of polyphenols and 45 previously identified compounds were detected [34].

Table 1 shows the compounds found in the 23 [3, 14, 20, 23, 24, 25, 28, 29, 30, 31, 33, 35, 36, 37, 38, 39, 40, 41, 42, 43, 44, 45, 46] selected studies regarding the phytochemistry profile of the plant (211 compounds). These include 72 phenolic compounds, 48 flavonoids, 60 terpenes, 16 FAs, 4 vitamins, 3 alkanes, 5 alkaloids, and 3 benzenoids. It can be seen that phenolic compounds were found most frequently among the studies, with chlorogenic acid (29) found in 10 studies, 3,5‐di‐O‐caffeoylquinic acid (18) found in 8 studies, and the compounds 4,5‐di‐O‐caffeoylquinic acid (27) and 3,4‐di‐O‐caffeoylquinic acid (15) found in 7 studies. Flavonoids and terpenes were also found in high diversity, indicating that sunflowers are rich in these classes.

**FIGURE 1:**
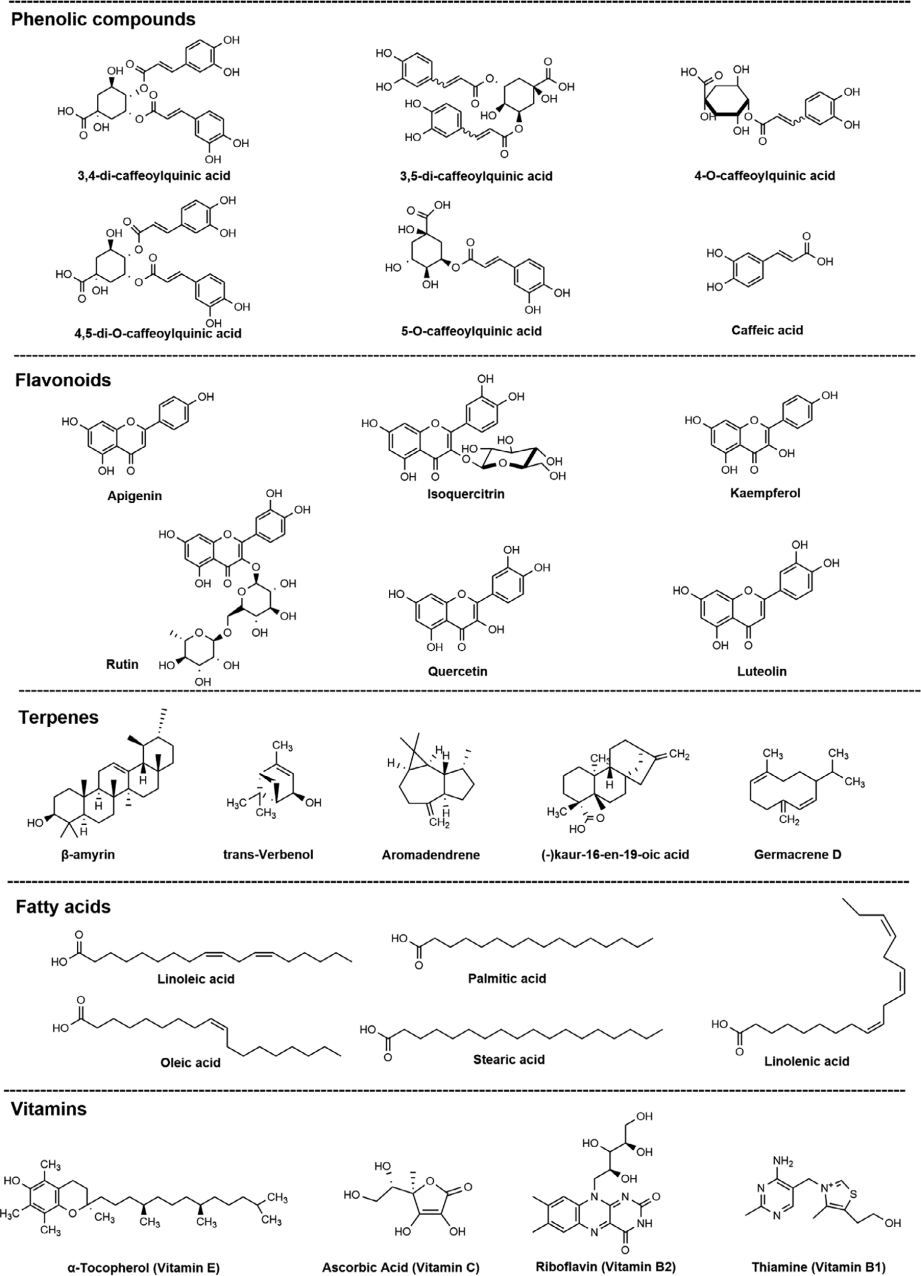
Chemical structure of the main compounds.

Figure 1 shows the chemical structures of the most common compounds among the phenolic compounds, flavonoids, terpenes, FAs, and tocopherols found.

#### 3.2.5 Chemical Space Analysis

**FIGURE 2:**
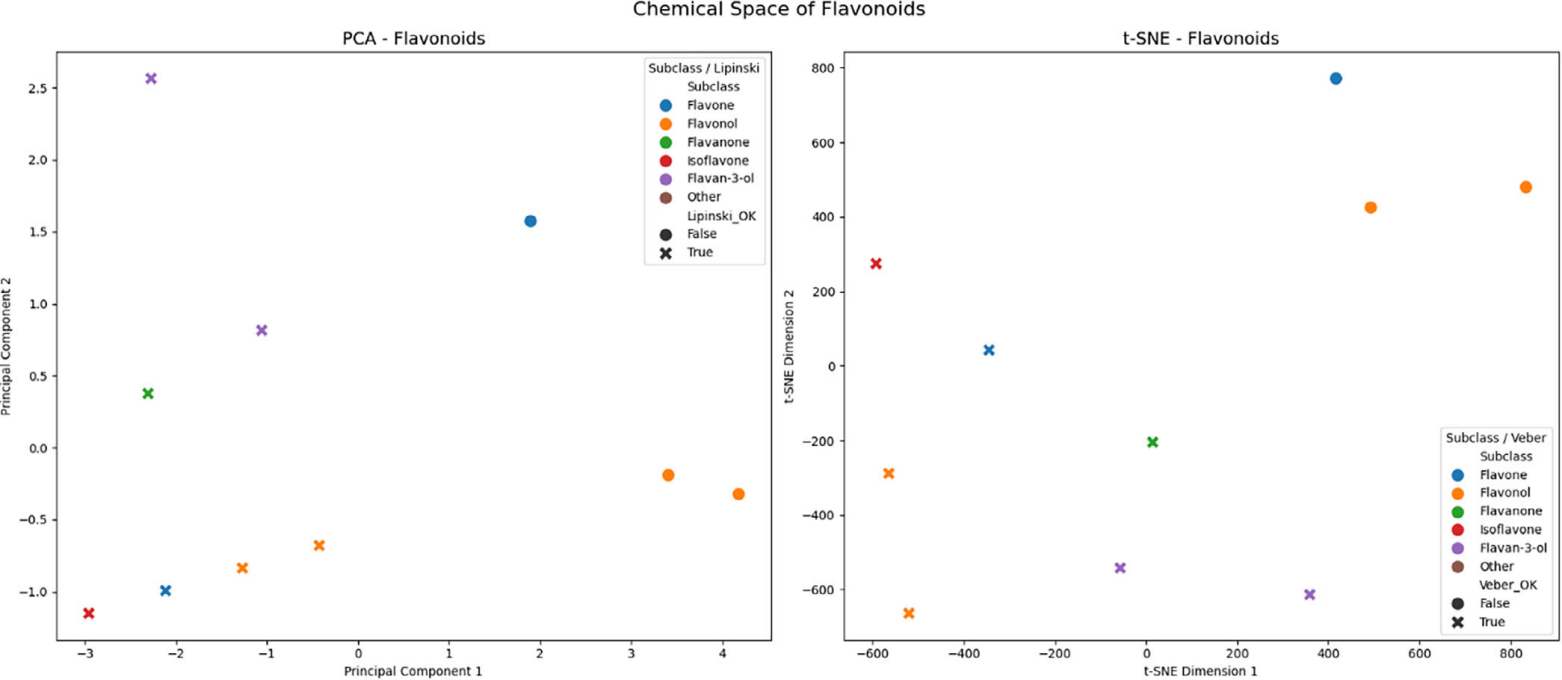
Chemical space maps of the flavonoids identified in *Helianthus annuus* L., representing their structural diversity and physicochemical properties. On the left, the principal component analysis (PCA) graph shows the distribution of compounds based on classical molecular descriptors, revealing patterns of similarity by subclass (color) and conformity with the rules of Lipinski (shape). On the right, the t‐distributed stochastic neighbor embedding (t‐SNE) graph represents the structural similarity based on molecular fingerprints, showing the formation of clusters by subclass and adherence to the rules of Veber. Colors represent chemical subclasses of flavonoids, while the shape of the markers indicates whether the compounds meet (circle) or do not meet (X) the drug‐likeness criteria according to Lipinski or Veber.

Chemoinformatic analysis revealed that the flavonoids identified in *H. annuus* L. show significant chemical diversity, with different distribution patterns in chemical space, both in the PCA and t‐SNE maps. Apigenin, luteolin, kaempferol, quercetin, naringenin, hesperidin, daidzein, catechin, rutin, and silibinin were used in the analysis. Compounds such as quercetin, luteolin and their glycosylated derivatives formed distinct clusters, indicating significant variations in physicochemical properties and structural similarity. The presence of characteristic subgroups, such as flavones and flavonols, was a determining factor in the organization of the clusters, especially in the fingerprint projection. In addition, three of the compounds analyzed (two flavonols and one flavone) met Lipinski and Veber's rules, reinforcing the potential of these flavonoids as promising candidates for oral administration in future pharmacological studies [47]. The choice to restrict this analysis to flavonoids is justified both by their abundance in HAEs and by their well‐documented pharmacological relevance. Flavonoids are known for their biological multifunctionality, acting on different molecular targets with antioxidant, anti‐inflammatory, antimicrobial and hepatoprotective effects [48, 49, 50]. As they are compounds widely distributed in the aerial parts of the plant and frequently associated with the medicinal activity of sunflowers, concentrating the analysis on them allowed a deeper and more targeted investigation into the constituents most likely to be therapeutically relevant. In addition, their structural diversity provides an excellent model for exploring patterns of similarity and molecular properties with potential for pharmaceutical application (Figure 2).

These results are particularly relevant in the context of the medicinal use of sunflowers, since flavonoids play a central role in the biological activities attributed to the species. The characterization of the chemical space not only confirms the structural heterogeneity of these metabolites, but also provides evidence to support their therapeutic use, especially due to the presence of compounds that meet drug‐likeness criteria. This suggests that the flavonoids of *H. annuus* are not only chemically distinct, but also pharmacologically viable, strengthening their value as a phytotherapeutic source and as raw material for the development of new drugs.

## 4 Pharmacological Properties

In the search, 23 studies regarding pharmacological properties were included: 9 studies were found reporting in vitro/in vivo antioxidant activity, 6 reporting anti‐inflammatory activity in rats and mice, 4 reporting antimicrobial activity, mainly antibacterial, four reporting hypoglycemic and hypolipemic, and 2 for nephroprotective and gastrointestinal activities (Table 2).

**TABLE 2:** Pharmacological properties of *Helianthus annuus* extracts, part of the plant used, and the mainly results observed.

Propriety	Method/model	Part of the plant	Treatment	Mainly results	References
Antioxidant	DPPH‐scavenging activity, ABTS and reducing power assay	Seeds	Extract fractions of 80% methanol	Four of six fractions of the sunflower seed extract (0.1 mg/assay) inactivated more than 90% of the DPPH radicals. The TEAC result for Fraction V was 2.21 mmol Trolox eq/g, and the ABTS reduction capacity was also higher for Fraction V. This fraction had the highest value of phenolic compounds	[23]
	Iron ion reduction capacity, ABTS and DPPH‐scavenging activity	Receptacles	Sunflower's receptacles extracts: petroleum ether fraction (PEF), ethyl acetate fraction (EAF), *n*‐butanol fraction (nBUF), and water fraction (WAF)	The DPPH and ABTS tests showed that the ethyl acetate fraction (EAF) exhibited strong activity between concentrations of 150–625 µg/mL, with results similar to those of the standard isoquercetin. The iron ion reduction capacity also showed that EAF was stronger. The reduction capacity correlated with the concentration of the fraction. EAF also had the highest flavonoid content in four fractions	[38]
	DPPH‐scavenging activity	Flower, bark, and leaves	Ethanolic (96%) extract of sunflower flowers, leaves, and bark	The DPPH method showed an IC_50_ of 48.84 for the leaves. Flower and bark extracts showed a weak antioxidant intensity	[51]
	DPPH‐scavenging activity, and reactive oxygen species (ROS) detection	Leaves	Absolute ethanolic extract of sunflower leaves	The ethanolic extract of *H. annuus* showed activity comparable to lycopene (a well‐established antioxidant), with 100 µg having the maximum radical scavenging activity compared to the standard ascorbic acid	[52]
	DPPH‐scavenging activity, and ferric reducing antioxidant power (FRAP)	Leaves	80% methanol extract of sunflower leaves (150–600 mg/kg)	FRAP showed an increase (*p* < 0.05) in total antioxidant power at 200 and 400 µg/mL of HAE, which was higher than that of ascorbic acid. HAE produced a concentration‐dependent increase in the DPPH assay, with optimal activity at 200 µg/mL	[53]
	SOD, CAT, and TBARS levels	Leaves	Hydromethanol extract of sunflower leaves (150, 300, and 600 mg/kg daily)	HAE reduced TBARS levels compared to the negative control (*p* < 0.05). SOD did not vary between groups (*p* > 0.05), and CAT was higher for the groups that received the treatments (*p* < 0.05)	[54]
	TEAC, DPPH‐scavenging activity and FRAP assays	Aerial parts	80% methanol extract of *H. annuus* leaves aerial parts in five growth stages	TEAC and FRAP increased (*p* < 0.05) between the initial and intermediate stages of flowering. DPPH scavenging activity was better in the intermediate flowering stage (*p* < 0.05) than in the visible bud stage. The EC_50_ values were 0.13 and 0.21 mg/mL, respectively	[30]
	DPPH‐scavenging activity, ABTS, FRAP, reducing power and oxygen radical absorbance capacity	Florets	Disc and ray florets extracts: ethyl acetate, 90% (v/v) aqueous ethanol, 90% (v/v) aqueous methanol, 50% (v/v) aqueous ethanol, 50% (v/v) aqueous methanol, or distilled water	Antioxidant activity was highest for aqueous alcohol solvents, followed by water and ethyl acetate. The best DPPH and reducing power results were obtained for 90% methanol extracts	[31]
	DPPH‐scavenging activity, and the iron‐reducing assay	Sprouts	80% aqueous methanol of sunflower sprouts (4–200 µg/mL)	HAE exhibited the most potent DPPH radical scavenging activity compared to other plant extracts (*L. palustris*, *V. angulariz*, and *T. sinensis*). For the reducing power assay, the addition of HAE to the reaction mixture resulted in a significant increase in the value	[40]
Anti‐inflammatory/analgesic	TPA‐induced mice	Petals	Triterpene glycosides isolated from an *n*‐butanol (n‐BuOH)‐soluble fraction of a MeOH sunflower petals extract	All extracted triterpene glycosides showed potent anti‐inflammatory effects (ID_50_ of 65–262 nmol/ear) compared to the control (*p* < 0.01), and more potent than indomethacin (ID_50_ = 838 nmol/ear), a commercially available anti‐inflammatory drug. Compared to hydrocortisone (ID_50_ = 83 nmol/ear), compound **6** (ID_50_ 65 nmol/ear) exhibited a similar effect	[44]
	MSU‐induced gouty arthritis rats	Head	Sunflower head extract (1 g/kg) with 0%, 20%, 40%, 60%, 80%, and 100% ethanol	The 20% ethanol extract (SHEB) showed the best inhibitory effects on swelling, reducing it by 16.2% in 12 h and 27.1% in 48 h (*p* < 0.05). SHEB reduced serum uric acid (UA) levels in mice with hyperuricemia by 50.0% (*p* < 0.05)	[32]
	Rats induced paws with formalin and egg albumin	Leaves	Methanol (80%) extract of sunflower leaves (150, 300, and 600 mg/kg)	The reduction in paw swelling was 33.33% for 300 and 600 mg/kg in the third hour of the experiment. The extracts significantly reduced paw swelling in the treated groups compared to the negative control (*p* < 0.05). For the egg albumin‐induced group, the reduction in edema volume was 32.94% and 31.76% for the 300 and 600 mg/extract groups, respectively. There was a significant difference compared to the control group (*p* < 0.05)	[55]
	Atopic dermatitis (AD) via modulation of T‐cell activity in mice	Leaves	Ethanol extract of sunflower leaves (100 and 250 mg/kg)	It has been confirmed that oral administration of HAE decreases the clinical score in mice with AD (*p* < 0.05). HAE reduces dermal and epidermal thickness on Days 14 and 28 (*p* < 0.05)	[14]
	TPA‐induced mice	Flowers	Three diterpene acids (grandiflorolic, kaurenoic, and trachylobanoic acids) isolated from a petroleum ether fraction of sunflower flowers	Topical treatments, especially with grandiflororic acid, reduced the extent of TPA‐induced swelling (*p* < 0.05). Pretreatment with diterpenoids reduced TPA‐induced MPO levels (*p* < 0.05)	[45]
	CVH mouse model of abdominal pain	Seeds	Cyclic peptide analgesic derived from sunflower (helianorphin‐19)	Helianorphin‐19 reduced the action potential of colonic nociceptors evoked by mechanical stimulation (*p* < 0.001)	[56]
Antimicrobiane	Grams‐positive *Staphylococcus*, *Streptococcus*, and *S. aureus*, and Gram‐negative *E. coli* and *Klebsiella*	Seeds and flowers	Flowers and seeds hexane, chloroform, ethyl acetate, butanol, water extracts (500, 250, 125, and 61.5 µg/mL)	The aqueous extract of the *H. annuus* flower showed better bacterial inhibition compared to the positive (levofloxacin) and negative control groups. The hexane extract inhibited all bacterial strains at all concentrations except *Streptococcus* at 125 and 62.5 µg/mL	[57]
	*Bacillus subtilis* and *Aliivibrio fischeri*	Leaves	Ethyl acetate sunflower leaves extract	The isolated compounds H1 (acid (−)kaur‐16‐en‐19‐oic) and H2 (acid 15‐α‐angeloyloxy‐ent‐kaur‐16‐en‐19‐oic) inhibited *B. subtilis* F1276 and *A. fischeri*	[28]
	*Streptococcus pyogenes* and *Streptococcus agalactiae*	Leaves	Ethanolic (absolut) extract of sunflower leaves (10–175 µg/mL)	The ethanolic extract of *H. annuus* inhibited *S. agalactiae* in a 20 mm clearance zone at 175 µg/mL compared to penicillin (21 mm). The extract, at a concentration of 125 µg/mL, presented a complete clearance zone as did Erythromycin for *S. pyogenes*	[52]
	In vitro antimalarial test with cultures of *Plasmodium falciparum* strain 3D7 and in vivo 36 BALB/c mice infected *P. berghei*	Roots, stems, seeds, flowers, and leaves	Ethanolic (96%) extract of sunflower roots, stems, seeds, flowers, and leaves (1, 10, 100, and 250 mg/kg)	Concentrations lower than 10 µg/mL inhibited the 3D7 strain of *P. falciparum*. Compared to the other parts, the root was more effective in terms of antimalarial effect in vitro (IC_50_ = 2.3 ± 1.4 µg/mL). The root extract also showed the best inhibition effect (63.6 ± 8.3%) against *Plasmodium berghei*. The % inhibition of *P. berghei* in mice was 72.3 ± 5.3 for 250 mg/kg of the extract	[58]
Hypoglycemic	Male albino Wistar rats induced to diabetes by alloxan	Leaves	Methanol (80%) extract of sunflower leaves (150, 300, and 600 mg/kg)	The extracts promoted a significant reduction in blood glucose levels compared to the control group (*p* < 0.05), reaching normal levels (< 100 mg/dL) of FBG on Day 21. HbA1c was also significantly (*p* < 0.05) lower for the treated groups	[54]
	Male albino Wistar rats induced to diabetes by alloxan	Leaves	Methanol (80%) extract of sunflower leaves (150, 300, and 600 mg/kg)	The extract in 600 mg/kg reduced the FBG 3 and 6 h after the treatment, when compared to the control group (*p* < 0.05)	[53]
	Healthy obese adults	Seeds	Capsule containing 250 mg of sunflower seed extract for 12 weeks	There was a reduction in blood glucose levels in the group using the extract (−3.71) compared to the control group (−2.61), but without significant difference (*p* > 0.05). HbA1c did not differ between the groups (*p* > 0.05)	[24]
Lipid‐lowering	Healthy obese adults	Seeds	Capsule containing 250 mg of sunflower seed extract for 12 weeks	The treatment reduced BMI and waist circumference (*p* = 0.02 and 0.001, respectively). Cholesterol levels were lower in the treatment group than in the placebo group (*p* < 0.05)	[24]
	Male albino Wistar rats induced to diabetes by alloxan	Leaves	Hydromethanol leaf extract of sunflowers leaves (150, 300, and 600 mg/kg daily)	The levels of total serum cholesterol, triglycerides, VLDL‐C, and LDL‐C in the control group rats were higher than in the rats treated with the extract (*p* < 0.05). HDL‐C was higher (*p* < 0.05) in the treated groups	[54]
	Adults aged between 19 and 65 years and a BMI of 25–31.9 kg/m^2^	Seeds	500 mg of sunflower seed extract plus 280 mg of additive substances, maltodextrin (112.5 mg), cellulose (110 mg), magnesium stearate (20 mg), hypromellose (20 mg), and silicon dioxide (15 mg)	Body fat mass, body weight, BMI and hip circumference significantly decrease in the sunflower extract treatment group (*p* < 0.05). Leptin and triglyceride decreased in the treated group (*p* < 0.05)	[59]
Colon‐protective	SPF male BALB/c mice induced to constipation by loperamide hydrochloride	Receptacles	Sunflower dietary fiber (SDF) extracted by citric acid extraction (ASDF) (0.25, 0.5, and 1 g/kg, for 8 days twice a day)	The ASDF group (0.5 and 1.0 g/kg) significantly reduced the time to first defecation with black stools (*p* < 0.05 and *p* < 0.01, respectively). Fecal weight 4 h after administration increased significantly in the treated groups (*p* < 0.05). In addition, treatment with ASDF showed improvement in colonic tissue damage	[60]
	DSS‐induced colitis in B6 mice	—	Sunflower polysaccharide (SP) prepared using a hydrogen‐peroxide‐assisted method	Intervention with SP led to relief of reduced colon length, but without statistical significance (*p* > 0.05)	[61]
Nephroprotective	Male albino Wistar rats	Leaves	Methanol (80%) extract of sunflowers leaves (150, 300, and 600 mg/kg)	The urea level was significantly lower (*p* < 0.05) in the groups treated with the extract doses. Serum creatinine did not differ between the groups (*p* > 0.05)	[62]
	Gentamicin induced nephrotoxic male mice	Seeds	Crude extract and hexane fraction of sunflower seeds (250 and 500 mg/kg ip)	HAE administration improved serum creatinine, urea and urea nitrogen levels (*p* < 0.01). In addition, treatment with *H. annuus* prevented gentamicin‐induced nephrotoxicity	[63]

Abbreviation: ABTS, 2,2′‐azino‐bis(3‐ethylbenzothiazoline‐6‐sulfonic acid); AD, atopic dermatitis; CAT, catalase; CVH, chronic visceral hypersensitivity; DPPH, 2,2‐diphenyl‐1‐picrylhydrazyl; DSS, dextran sulfate sodium; FBG, fasting blood glucose; FRAP, ferric reducing antioxidant power assay; HbA1c, glycosylated hemoglobin; HAE, *H. annuus* extract; MPO, myeloperoxidase; MSU, monosodium urate; SOD, dismutase superoxide; TBARS, thiobarbituric acid reactive substances; TEAC, Trolox equivalent antioxidant capacity; TPA, 12‐O‐tetradecanoylphorbol‐13‐acetate.

The seeds were reported in all pharmacological activities described, with the exception of anti‐inflammatory and gastroprotective activity. It is necessary to investigate seed extract in inflammation, since it contains saponins that may have anti‐inflammatory potential [6]. The leaves were the part of the plant most studied for pharmacological properties; a greater number of studies were found on antioxidant activity, which showed an important effect, but also on antimicrobial, anti‐inflammatory, metabolic, and nephroprotective effects. For the flowers, antioxidant, antimicrobial, and anti‐inflammatory effects were found, requiring further investigation into metabolic disorders. The extracts from the receptacles showed antioxidant activity, and their isolated fibers showed a colon‐protective effect. One study found antimalarial potential in the ethanol root extract of *H. annuus*.

### 4.1 Toxicology Assessment

Six studies reported on the toxicity and safety of the doses of extracts administered. In rats, doses of 300–3600 mg/kg of leaf extract proved tolerable, with no deaths or clinical signs of toxicity within 48 h of observation. However, unformed or malformed feces appeared between 6 and 12 h after treatment with doses of 2400 and 3600 mg/kg [53]. Another study with rats showed no change in body weights and organ indices in the experimental animals, suggesting the safety of the extract at a dose of 1 g/kg [32]. The study by Díaz‐Viciedo et al. found no cytotoxic effect of the three diterpenic acids isolated from sunflower at concentrations of 1–20 µM in rats [45]. The study by Ekasari et al. observed a low average survival time at 800 mg/kg of the sunflower root extract, suggesting that the extract at this dose tends to be toxic [58]. For mice, no toxicity or mortality was found after 24 h of treatment with doses of 3 g/kg (ip) and 15 mL/kg (ip) of the seed extract [63]. In humans, no changes were observed in the participants' vital signs, biochemical analyses, physical examinations, or ECGs, but constipation occurred in 11 participants in the sunflower group and 5 in the placebo group [24].

### 4.2 Antioxidant Activity

The antioxidant properties of *H. annuus* have been highlighted, probably due to the plant's rich phenolic content. Karamać et al. [23] evaluated the antioxidant capacity of *H. annuus* kernels in the DPPH‐scavenging activity test and found inactivation of more than 90% of DPPH radicals at a concentration of 0.1 mg/assay of the extract fractions [23]. The highest reducing power and ABTS were noted for Fraction V, which had the highest presence of phenolic compounds and 5‐CQA. The authors suggest that the significant presence of 5‐CQA may be directly involved in capturing free radicals and inhibiting lipid oxidation. This compound is a chlorogenic present in many fruits, herbs, and vegetables and is highlighted due to its important antioxidant activity [64].

The receptacles of *H. annuus* were also investigated by Qiao et al. [38]. The iron ion reduction capacity, ABTS, and DPPH tests were carried out to evaluate the antioxidant activity. The free radical scavenging activities of all the samples under the concentration gradient of 0–625 µg/mL, ethyl acetate fraction (EAF) showed higher free radical scavenging activity in DPPH assay and the best scavenging ability in ABTS, in similar isoquercetin concentrations. The iron ion reduction abilities of EAF were also stronger concerning other fractions, and the reduction ability became stronger as the concentration increased. This must be because EAF had the highest flavonoid contents, including isoquercetin [38].

For the *H. annuus* leaves, the DPPH method showed an IC_50_ of 48.84, and the flower and stem extracts showed a DPPH result of 180.50 and 274.02, respectively, both with low antioxidant activity [51]. The leaf extract has higher phenolic levels (35.149 mg of GAE/g of extract), and also a higher amount of flavonoids (10.92 + 1.3) [51]. This can be a possible reason for the best antioxidant activity shown by the leaf extract. The study by Alshahrani et al. evaluated the ethanolic extract of *H. annuus* leaves, finding good activity in comparison with lycopene, which is an antioxidant known for its potency [52]. Other study indicates the *H. annuus* leaf extract as an antioxidant agent, showing a concentration‐dependent increase in antioxidant activity (%) in the DPPH assay, with the best activity observed at 200 µg/mL, a good effect compared to vitamin C. The FRAP test showed a significant increase in the total antioxidant power of the extract at doses of 200 and 400 µg/mL, a better result than vitamin C [53]. Gai et al. found that the highest antioxidant activity of sunflowers was generally determined in older plants (mid and late flowering stages), with phenolic compounds being correlated with the antioxidant activity [30].

*H. annuus* florets showed a good antioxidant activity [31]. The antioxidant tests showed that both the Rf and Df extracts were most promising for the hydroalcoholic extracts, followed by the aqueous and ethyl acetate. This suggests a direct correlation between the presence of phenolic compounds and antioxidant activity. The 90% methanol extracts for both the Rf and Df were the highest among the other extracts for DPPH and ABTS in both samples [31].

Finally, Sun et al. evaluated the antiglycative and antioxidant effects of *H. annuus* sprouts [40]. HAE generated a great potent capture activity in the DPPH assay, and the iron‐reducing assay showed that the addition of HAE resulted in a markedly increased value, suggesting its significant iron‐reducing potential. In addition to its antioxidant effects, HAE also inhibited the advanced glycation end‐products (AGEs) formation at a concentration of 0.2 mg/mL, with an inhibitory rate of 37.23%. At a concentration of 1.0 mg/mL, *H. annuus* obtained an inhibition rate of 83.29%. This result shows an inhibition rate higher than AG (1 mM), a well‐known synthetic AGE inhibitor, which has an inhibitory rate of 80.88%. These activities must be related to cynarin (1,5‐dicaffeoylquinic acid) in the extract, calculated at around 8% [40]. The sunflower husk extract also shows an important activity in the elimination of reactive oxygen species (ROS) when evaluating the protection of *Arabidopsis*, which is a sensitive method for evaluating agents that damage the DNA of this species [34]. The antioxidant activity of sunflowers may be due to the presence of polyphenols and other bioactive compounds, such as flavonoids and phenolic acids.

### 4.3 Anti‐Inflammatory and Immunomodulatory Activity

Some studies have also evaluated the anti‐inflammatory and immunomodulatory capacity of *H. annuus*. The anti‐inflammatory effects of the *H. annuus* petals extract were investigated in TPA‐induced mice, using hydrocortisone and indomethacin as positive controls. Two triterpene glycosides (helianthoside 4 and 5), and four triterpene glycosides (helianthosides 1, 2, 3, and B), were isolated from an *n*‐butanol‐soluble fraction of a methanol extract of *H. annuus* petals [44]. All six isolated compounds showed significant inhibitory effects on inflammation, generating more promising effects than indomethacin (ID_50_ 65–262 nmol/ear vs. ID_50_ = 838 nmol/ear) [44]. Another study, using MSU‐induced gouty arthritis rats, found that the sunflower head extract (SHE) in concentrations of 0%–60% ethanol suppressed the inflammation after 12 h of induction, and SHEA (0% ethanol) and SHEB (20% ethanol) were effective after 48 h [32]. SHEB had the best inhibitory effects on swelling, reducing it by 16.2% in 12 h and 27.1% in 48 h (*p* < 0.05). SHEB also had the best suppressive effect on serum uric acid (UA) levels in mice with hyperuricemia, reducing the serum UA level by 50.0% (*p* < 0.05) [32]. The authors found that treatment with the extract reduced levels of xanthine oxidase (XO), an important enzyme in the synthesis of UA, in liver tissue and serum. In addition, malondialdehyde (MDA), an important marker of oxidative stress, also decreased. The SHEB showed good concentrations of protein, polysaccharides, reducing sugars, and flavonoids compared to extracts with a higher concentration of ethanol [32]. However, the study did not report the TPC, which could be a determinant of the observed effects in SHEB.

A study by Onoja et al. (2019) [55] evaluated the analgesic and anti‐inflammatory activity of the methanolic extract of *H. annuus* leaves in rats with induced paw edema with formalin and egg albumin. At the third hour of the experiment, the formalin‐induced paw reduction was 36.36% for the acetylsalicylic acid (ASA) positive control and 33.33% for both 300 and 600 mg/kg [55]. The extracts significantly reduced paw volume in the treated groups compared to the negative control (*p* < 0.05). For the egg albumin‐induced group, the reduction in edema volume was 35.29% for ASA and 32.94% for the 300 mg/kg extract group [55]. As for the analgesic activity induced by acetic acid, the treatment groups demonstrated a significant improvement in nociception (*p* < 0.05). For the tail‐flick test, the reaction time to pain was greater in the positive control (pentazocine) and extract groups, showing a significant response compared to the control group (*p* < 0.05) [55]. The effects of *H. annuus* leaf extract were also evaluated on atopic dermatitis (AD) via modulation of T‐cell activity in mice [14]. The group that received oral administration of the extract showed better characteristics (skin enlargement and ear swelling) in a dose‐dependent manner. The 250 mg/kg extract performed better on Days 14 and 28 after AD induction. The authors suggest that *H. annuus* leaves have immunomodulatory potential in AD by regulating T‐cell activity [14]. In addition, the extract was able to significantly reduce serum immunoglobulin (Ig)‐E levels, making it a possible alleviator of AD symptoms [14]. These results suggest that *H. annuus* leaf extract can be used as an agent to improve inflammatory conditions and promote analgesia.

**FIGURE 3:**
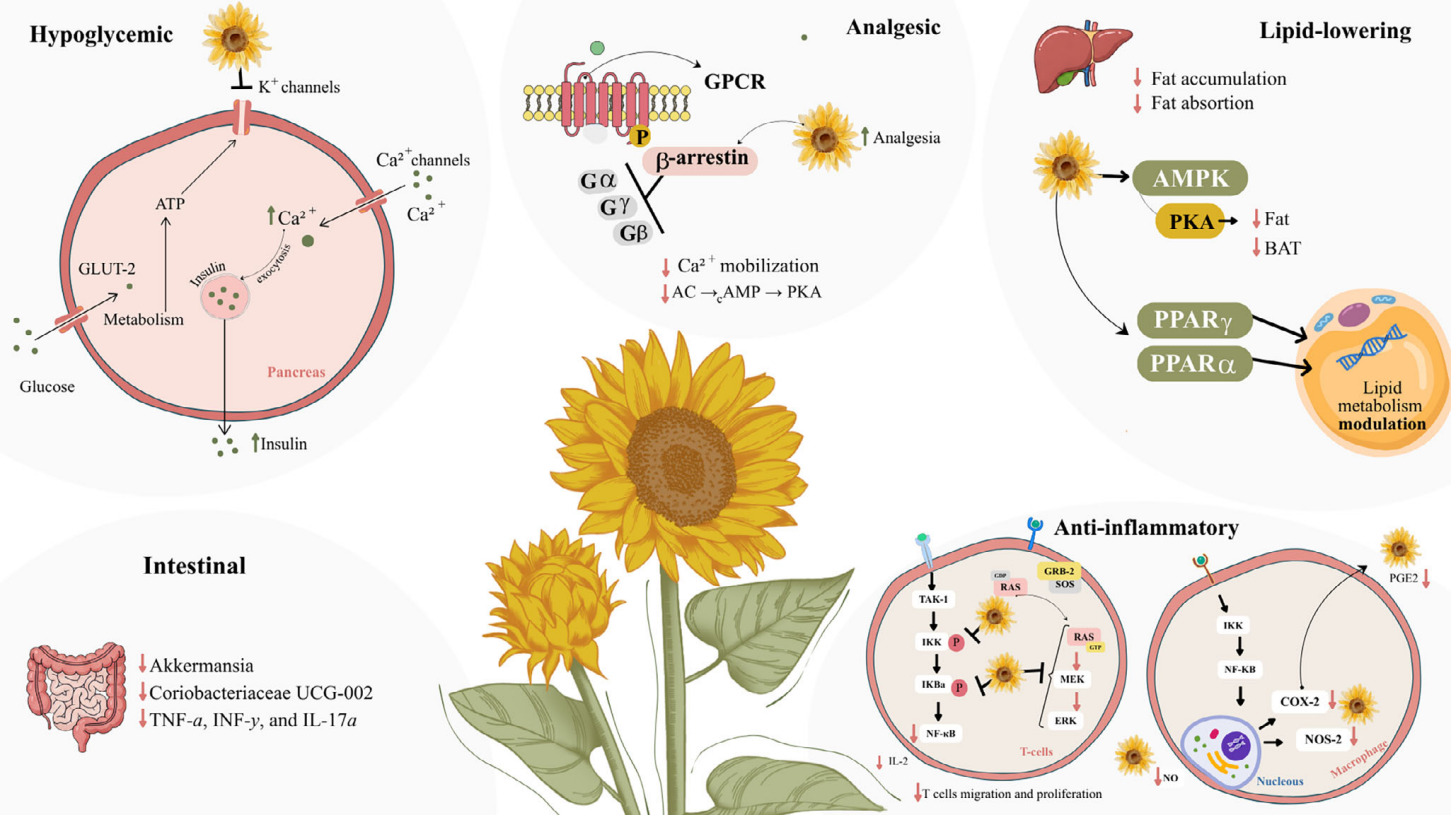
Possible cellular mechanisms of action involved in the therapeutic effects of *Helianthus annuus* extracts. Created by Jessiane Bispo de Souza and Marina dos Santos Barreto with Canva. Copyright 2026, Canva Pty Ltd.

The *H. annuus* flowers also showed a modulatory response to inflammation [45]. Three isolated diterpenoids were evaluated for anti‐inflammatory activity: Grandiflora acid, kaurenoic acid, and thachylobanoic acid. These compounds inhibited the release of TNF‐α in a concentration‐dependent manner and also reduced the release of nitric oxide (NO) and prostaglandin (PGE‐2), which are pro‐inflammatory mediators [45]. An in vivo test was conducted on the TPA‐induced mouse ear edema model. The three diterpenoids were applied topically and significantly reduced the extent of TPA‐induced swelling and the accumulation of myeloperoxidase (MPO) activity. In addition, Type 2 isoform of nitric oxide synthase (NOS‐2) inhibition and reduced cyclooxygenase (COX‐2) enzyme expression were observed [45]. These enzymes play an important role in inflammation conditions, and their reduction can suggest an inflammation containment [65]. Another study found that annuolide A‐15‐O‐β‐d‐glucopyranoside, a sesquiterpene lactone isolated from *H. annuus* leaves aqueous extract, showed significant inhibition of NO secretion, contributing to an anti‐inflammatory action [41]. Figure 3 represents this anti‐inflammatory mechanism.

Meng et al. [66] also show that *H. annuus* stem polysaccharides have an inhibitory potential of cancer proliferation and metastasis via TNF‐α [66]. The group of mice treated with the polysaccharide (HSPP‐4) subfraction of the stem had a survival rate of 50.7 ± 12.7 compared to the control group, which had a survival of 38.7 ± 12.7 (*p* > 0.01) [66]. The HSPP4 fraction also showed effects on inhibiting tumor cell metastasis in that it was able to reduce TNF‐α in macrophages [66].

### 4.4 Antimicrobial and Larvicidal Potential

In addition to its antioxidant, anti‐inflammatory, and immunomodulatory properties, some studies have highlighted the antimicrobial and larvicidal activity of *H. annuus*. The *H. annuus* flowers' antimicrobial activity was also tested against five pathogenic microorganisms: Gram‐positive *Staphylococcus*, *Streptococcus*, and *S. aureus* and harmful Gram‐negative bacteria *Escherichia coli* and *Klebsiella* [57]. The aqueous *H. annuus* flower extract showed better bacterial inhibition compared to positive (levofloxacin) and negative control groups. The hexanic extract significantly inhibits all the bacterial strains at all concentrations, except *Streptococcus* at 125 and 62.5 µg/mL. The methanolic extract also provided potent inhibition against all bacterial strains at all concentrations [57].

Another study highlight the sunflower's leaves diterpenes ((−)kaur‐16‐en‐19‐oic acid and 15‐α‐angeloyloxy‐ent‐kaur‐16‐en‐19‐oic acid) in inhibiting *Bacillus subtilis* and *Aliivibrio fischeri* [28]. Compound H3 isolated from the *H. annuus*, although not identified, showed good antibacterial effects against *B. subtilis* [28]. Regarding the antibacterial efficiency of the ethanolic extract of *H. annuus* leaves on resistant bacterial strains (*Streptococcus pyogenes* and *Streptococcus agalactiae*), the results showed that the extract, alone or added to lycopene, was able to inhibit *S. agalactiae* at 175 µg, and can be compared with penicillin. Furthermore, at a concentration of 125 µg, the extract showed a complete clearance zone for *S. pyogenes*, similar to the positive control erythromycin [52].

The study by Ekasari et al. (2019) evaluated the antimalarial potential of parts of *H. annuus* [58]. The SFEs were prepared using roots, stems, seeds, flowers, and leaves. The in vitro antimalarial test was carried out with cultures of *Plasmodium falciparum* strain 3D7 (chloroquine‐sensitive). Doses lower than 10 µg/mL were observed to promote IC_50_ inhibition of *P. falciparum* strain 3D7. The root extract was the most effective in terms of the in vitro antimalarial effect (IC_50_ = 2.3 ± 1.4 µg/mL), followed by the leaves (IC_50_ = 4.3 ± 2.2 µg/mL) and the stem (IC_50_ = 4.8 ± 0.0 µg/mL). For the inhibition of *Plasmodium berghei*, the root extract also showed the best inhibition effect (63.6 ± 8.3%), followed by the leaves (59.3 ± 13.2) and the stem (40.3 ± 8.2) [58]. The authors also conducted an in vivo experiment, where 36 BALB/c mice were infected with a solution containing *P. berghei*‐infected enterocytes. The mice were separated into groups treated with 1, 10, 100, and 250 mg/kg of the root extract and the untreated group. The results show that mice treated with the root extract had an ED_50_ of 10.6 ± 0.2 mg/kg against *P. berghei*. An EC_50_ < 100 mg/kg indicates a great antimalaric activity. The % inhibition of *P. berghie* in mice was 72.3 ± 5.3 for 250 mg/kg of the extract [58]. The authors found that the ethanolic (96%) extract from the roots of *H. annuus* has as its main antimalarial mechanism the inhibition of heme detoxification, with better results than the standard drug, chloroquine. This is an important finding, given that malaria has major impacts in endemic regions, with more than 250 million cases recorded in 83 different countries by 2023 [67].

### 4.5 Hypoglycemic, Antidyslipidemic, and Hepatoprotective Effects

One of the traditional uses of sunflowers is its hypoglycemic and antidyslipidemic effects. Onoja et al. (2018) [54] investigated the hypoglycemic and hepatoprotective potential of the hydromethanolic extract of *H. annuus* leaves in diabetes‐induced rats. The extracts (150, 300, and 600 mg/kg) and glibenclamide (2 mg/kg) were significant in reducing blood glucose compared to the control group (*p* < 0.05) [54]. As for liver function, the treated groups had significant reductions in AST, ALT, and ALP levels compared to the control group (*p* < 0.05) [54]. Another study by Onoja et al. (2014) [53] evaluated the antidiabetic potential of the methanolic extract of *H. annuus* leaves in alloxan diabetes‐induced rats. The extract showed a dose‐dependent response in reducing fasting blood glucose (FBG). The 600 mg/kg extract significantly reduced FBG in rats 3 and 6 h after the experiment in comparison with the control group (*p* < 0.05) [53]. For hyperglycemic rats, 120 min after glucose administration, the control group had higher blood glucose compared to the treatments (*p* < 0.05) [53].

Leverrier et al. (2019) evaluated the effect of sunflower seed extract on anthropometric measurements, weight and biochemical parameters of obese adults (*n* = 50) in a hospital in Spain over 12 weeks [24]. The authors found that the group taking sunflower hydroalcoholic extract capsules (500 mg/kg/day) reduced BMI (*p* < 0.05) and waist circumference (*p* < 0.01) compared to the control group [24]. The reduction in cholesterol was also greater in the group using the extract (−18.43 mg/dL) compared to the control group (−8.72 mg/dL) (*p* < 0.05). In addition, there was a reduction in blood glucose in the group using the extract (−3.71) compared to the control group (−2.61) (*p* > 0.05) [24]. The authors suggest that this lipid‐lowering effect occurs through interaction with peroxisome proliferator‐activated receptors (PPAR)‐α and ‐γ, in addition to regulating lipid metabolism via the AMP‐activated protein kinase (AMPK) pathway [24] (Figure 3). Another placebo‐controlled trial conducted by Kim et al. (2024) [59] evaluated supplementation with sunflower seed extract (500 mg/kg) in adults with obesity. The results showed that daily administration (for 12 weeks) of the extract reduced body fat and improved hip circumference, body mass index, and body weight. No adverse effects were reported [59].

In the Onoja et al. [54] study, the values for cholesterol, triglycerides, VLDL‐c, and LDL‐c were lower in the SFE‐treated groups, and HDL‐c was higher in the groups treated with 300 and 600 mg/kg of the extract (*p* < 0.05). Histopathology of the pancreatic tissue showed a dose‐dependent increase in the number and size of pancreatic islands compared to the control group [54].

Rehman et al. [68] reported on the activities of sunflower seeds in diabetes, indicating that sunflower has significant potential to normalize blood glucose in humans and animals. Sunflower probably has an effect on increasing insulin secretion by β cells and by inhibiting glucose‐6‐phosphate translocase, which converts glucose‐6‐phosphate into glucose in the Krebs cycle [68]. In addition, an in vitro study showed that the extract of *H. annuus* leaves inhibits the enzymes α‐glucosidase and α‐amylase in a non‐competitive and competitive manner, respectively [69]. These enzymes are directly involved in the process of glucose absorption and can reduce hyperglycemic peaks resulting from feeding [69]. A systematic review study with meta‐analysis published by our group recently reported that SFEs have significant hypoglycemic potential, which may be due to their action in stimulating GLP‐1 release and regulating glucose absorption [70]. Then, these responses can be responsible for hypoglycemic and antilipidemic sunflower effects. The studies included in this review suggest that SFE acts similarly to glibenclamide, blocking the ATP‐sensitive potassium channel in the β cells, stimulating insulin release through calcium [53, 54] (Figure 3).

### 4.6 Gastrointestinal System Effects

Gastrointestinal beneficial effects of *H. annuus* use have also been reported. A study by Zhu et al. evaluated the constipation‐relieving potential in mice of soluble dietary fiber (SDF) from *H. annuus* receptacles [60]. Male SPF BALB/c mice were used for constipation induction by loperamide (1 mg/mL) by gavage for 16 days. The SDF from sunflower receptacles obtained by the citric acid extraction method (ASDF) significantly reduced the mice's defecation time from the first evacuation of black feces (*p* < 0.05) at different doses, with the highest dose being more effective (*p* < 0.01) [60]. In addition, the fecal weight of the mice 4 h after administration increased significantly in the low (0.25 g/kg), medium (0.50 mg/g), and high‐dose (1.0 g/kg) ASDF groups (*p* < 0.05) compared to the control group. With the histology of the colon, the authors suggested that ASDF has a certain repair function in the destruction of colon tissue caused by constipation and may play a role in protecting the colon [60]. Another study evaluated sunflower acid polysaccharide (SP) in improving colitis in male C57BL/6J (B6) mice. B6 mice were separated into control [61], dextran sodium sulfate (DSS)‐induced colitis, and treatment groups for 28 days. The group treated with SP experienced relief from the shortened length of the colon (*p* > 0.05) [61]. In addition, the study by Muratspahić et al. evaluated an analgesic stable cyclic peptide derived from sunflower seeds (helianorphin‐19) possibly involved in the treatment of chronic abdominal pain [56]. The cyclic peptide significantly reduced the visceromotor response (VMR) and significantly reduced the firing of the action potential of colonic nociceptors (*p* < 0.05) [56]. The authors suggest that helianorphin‐19 may modulate β‐arrestin‐2, which has desensitizing effects on G protein‐coupled receptor (GPCR) and leads to prolonged analgesia (Figure 3). These studies indicate that sunflowers may have a good response in treating gastrointestinal diseases, especially its fiber and polysaccharides, which can be used as an alternative treatment for people living with colon diseases.

### 4.7 Other Pharmacological Activities

Other pharmacological activities were reported for *H. annuus* use. The study by Onoja et al. [62] evaluated the effects of *H. annuus* against nephrotoxicity and cardiac and hematological diseases in rats induced to diabetes by alloxan. The HAE had a significant effect on weight gain during the treatment period compared to the control group (*p* < 0.05) [62]. As for the hematological patterns, no differences were found between the groups regarding PCV, red blood cells, hemoglobin, and MCV. MCH and MCHC were lower in the treated and positive control groups than in the negative control (*p* < 0.05) [62]. As for renal markers, urea levels were lower in the glibenclamide and extract groups compared to the control group (*p* < 0.05), there were no differences in creatinine (*p* > 0.05) [62]. The authors suggest that these effects were promoted by the phytoconstituents of *H. annuus* (flavonoids, tannins, alkaloids, glycosides, and saponins). In addition, Ali et al. [63] evaluated the nephroprotective effects of the methanolic and hexanoic extract of *H. annuus* seeds in mice‐induced nephrotoxicity with gentamicin. The administration of HAE in combination with gentamicin was able to reduce the levels of the renal markers urea, serum creatinine, and blood urea nitrogen (*p* < 0.01) [63]. This makes *H. annuus* an important medicinal plant, with its extract a possible alternative to treat kidney diseases.

Figure 3 shows the possible mechanisms of action involved in the hypolipidemic, anti‐obesity, anti‐inflammatory, analgesic, and gastrointestinal effects of HAEs and metabolites. Sunflower leaf extracts can promote glycemic lowering activities by blocking potassium channels in β cells, leading to the stimulation of calcium intake and insulin vesicle secretion [53, 54]. For analgesic purposes, the compound in sunflowers (helianorphine‐19) can lead to impaired β‐arrestin recruitment, which is responsible for desensitizing GPCR subunits, thereby prolonging analgesia [56]. For lipid‐lowering effects, sunflower seed extract can act by reducing the accumulation and absorption of lipids by the liver, as well as acting on PPAR‐α and ‐γ and decreasing fat and brown adipose tissue (BAT) production by activation of liver AMPK pathway [24]. The intestinal properties are related to sunflower polysaccharide, which acts by reducing harmful microorganisms, such as Akkermansia and Coriobacteriaceae, in addition to reducing TNF‐α, IL‐17α, and IFN‐y, pro‐inflammatory cytokines [61]. Finally, the anti‐inflammatory effects of sunflower leaf extract occur through the inhibition of IKK and IKBa phosphorylation, reducing NF‐κB, and inhibiting the MAPK pathway [14], in addition to the inhibition of COX‐2 and NOS‐2 by diterpenes, important enzymes in the inflammatory process [45].

### 4.8 Limitations and Future Directions

Despite existing studies, there is still a need for in‐depth phytochemical research on parts such as roots and leaves. In addition to investigating the existence of terpenes in sunflower seeds. It is also necessary to conduct more preclinical studies to investigate the possible pharmacological pathways involved in the observed therapeutic effect. The need for further clinical studies has also been identified, both to investigate the toxicity of the extracts and to evaluate the observed pharmacological effects and whether there was real translationality with the preclinical studies.

## 5 Conclusions

The presence of bioactive compounds such as phenolic acids (chlorogenic acid, caffeic acid, 4‐O‐caffeoylquinic acid, 3,4‐di‐O‐caffeoylquinic acid and 3,5‐di‐O‐caffeoylquinic acid), flavonoids and terpenes makes sunflower plant with important pharmacological potential. Its pharmacological impacts cover the endocrine, intestinal and renal systems. A greater number of important results were found for antioxidant and anti‐inflammatory activities, but their antimicrobial, hypoglycemic and lipid‐lowering activities also stand out. As for toxicity, pre‐clinical studies reported adverse effects at doses of 800 mg/kg for the root extract and 2400 and 3600 mg/kg for the leaf extract. A clinical study reported constipation at doses of 500 mg/kg/day of the seed extract. However, more studies are needed to assess the toxicity of HAEs and their correlation with the parts extracted. With this review, we highlight the importance of phytochemical research into sunflowers and its possible implications for humans. We emphasize that there is a need for more studies on this plant in humans and animals to investigate the pharmacological effects and toxicity of the plant's extracts. In addition, studies investigating the mechanisms of action of the activities provided by the extracts are necessary.

## Author Contributions

**Marina dos Santos Barreto**: conceptualization, methodology, data curation, investigation, writing – original draft. **Wesley Lisboa de Jesus**: conceptualization, methodology, data curation, writing – original draft. **Maria Eduarda de Britto Sá**: methodology, data curation, investigation, writing – original draft. **Jessiane Bispo de Souza**: data curation, investigation, writing – original draft. **Ronaldy Santana Santos**: data curation, investigation, writing – original draft. **Júlia Santana Lisboa**: data curation, investigation, writing – original draft. **Pedro Henrique Macedo Moura**: data curation, writing – original draft. **Deise Maria Rego Rodrigues Silva**: data curation, writing – original draft. **Eloia Emanuelly Dias Silva**: data curation, writing – original draft. **Lysandro Pinto Borges**: conceptualization, writing – review and editing. **Adriana Gibara Guimarães**: conceptualization, writing – review and editing.

## Conflicts of Interest

The authors declare no conflicts of interest.

## Supporting information




**Supporting File 1**: Table S1: Authors, type of extract, extraction method, phytochemical analysis and analysis method. Figure S1: Python codigs used for Chemical space analysis.

## Data Availability

Data sharing is not applicable to this article as no new data were created or analyzed in this study.
