# Supplementary material for: Therapeutic Activities and Phytochemical Composition of Helianthus annuus L. Extracts
Source: Chem Biodivers. 2026 Feb 16;23(2):e02471. doi: 10.1002/cbdv.202502471 (PMC12908930; doi:10.1002/cbdv.202502471)

Table S1: Authors, type of extract, extraction method, phytochemical analysis and analysis method.

| **Authors** | **Extract** | **Extraction Method** | **Part of the plant** | **Method used for analysis** |
| --- | --- | --- | --- | --- |
| Amakura et al. (2013) | 50% ethanol | Liquid-liquid extraction after homogenization | Seeds | RP-HPLC |
| Amakura et al. (2024) | Petroleum ether | Soxhlet | Seeds | NMR Spectroscopic |
| Díaz-Viciedo et al. (2008) | Methanol (0:100, 30:70, and 100 : 0 gradient) | Column chromatography | Flowers | (RP)-HPLC |
| Karamać et al. (2012) | 80% methanol | Solid-liquid extraction assisted by stirring and heating | Seeds | HPLC–MS(ESI) |
| Leverrier et al. (2019) | Hidroalcoholic (non-specified solvent) | Commercially acquired | Seeds | HPLC |
| Paja̧k et al. (2014) | 99.8% methanol | Solid-liquid extraction by stirring | Seeds | HPLC UV detection |
| Lee, Kim, and Jeong (2022) | Ethanol | Hydroethanolic extract by hot maceration | Leaves | HPLC-MS |
| Gai et al. (2020) | 80% methanol | Heat-assisted methanolic extraction with vacuum solvent removal and lyophilization | Aerial parts (from stem extension to late ﬂowering) | HPLC–DAD |
| Zoumpoulakis et al. (2017) | n-hexane | Ultrasonic Assisted Extraction (UAE) | Seeds | UHPLC-ESI-MS |
| Qiao et al. (2021) | Water, petroleum ether, ethyl acetate, n-butanol | Fractional liquid-liquid extraction | Receptacles | UHPLC-HRMS/MS |
| Ramparadath, Balogun and Sabiu (2023) | Essential oil | Soxhlet extraction using  petroleum ether (40/60 strength) | Seeds | GC–FID/FAMEs |
| Shakya and Bhatla (2010) | Chloroform/methanol (2:1) and n-hexane/isopropanol (3:2) | Lipid extraction using organic solvents | Pollen and stigma | CG |
| Ye et al. (2015) | Ethyl acetate, ethanol, methanol and water in different polarity | Liquid-liquid extraction with sonication | Ray florets and disc florets | RP-HPLC-DAD/ESI-TOF-MS |
| Liang et al. (2013) | 80% chilled acetone | Liquid-liquid extraction | Ray florets and disc florets | RP-HPLC-DAD/ESI-TOF-MS |
| Sun et al. (2012) | 80% methanol | Liquid-solid extraction | Sprouts | HPLC and LC–MS/MS |
| Bai et al. (2023) | Water extract (distilled water) | Aqueous extraction | Flower heads | HR-ESI-MS |
| Weisz, Kammarer and Carle (2009) | 80% methanol | Soxhlet extraction using n-hexane | kernels and shells | HPLC-MS(ESI) |
| Abbaschian and Soltani (2025) | 99.9 % ethanol | Organic solvent extraction | Petal | GC-MS |
| Özcan et al. (2024) | Methanol | Liquid-liquid extraction with sonication | Seeds | HPLC |
| Fuentes-Gandara et al. (2019) | Ethanol in water from 0 to 100% | High-pressure techniques | Leaves | HPLC and CC |
| Abdalla et al. (2021) | Methanol | Maceration | Seeds | LC–ESI–MS/MS |
| Móricz et al. (2018) | Ethyl acetate | Maceration | Leaves | HPTLC-MS |
| Ukiya et al., 2007 | n-butanol-soluble fraction of a methanol extract | Immersion | Flower petals | NMR Spectroscopic |

Figure S1: Python codigs used for Chemical space analysis.


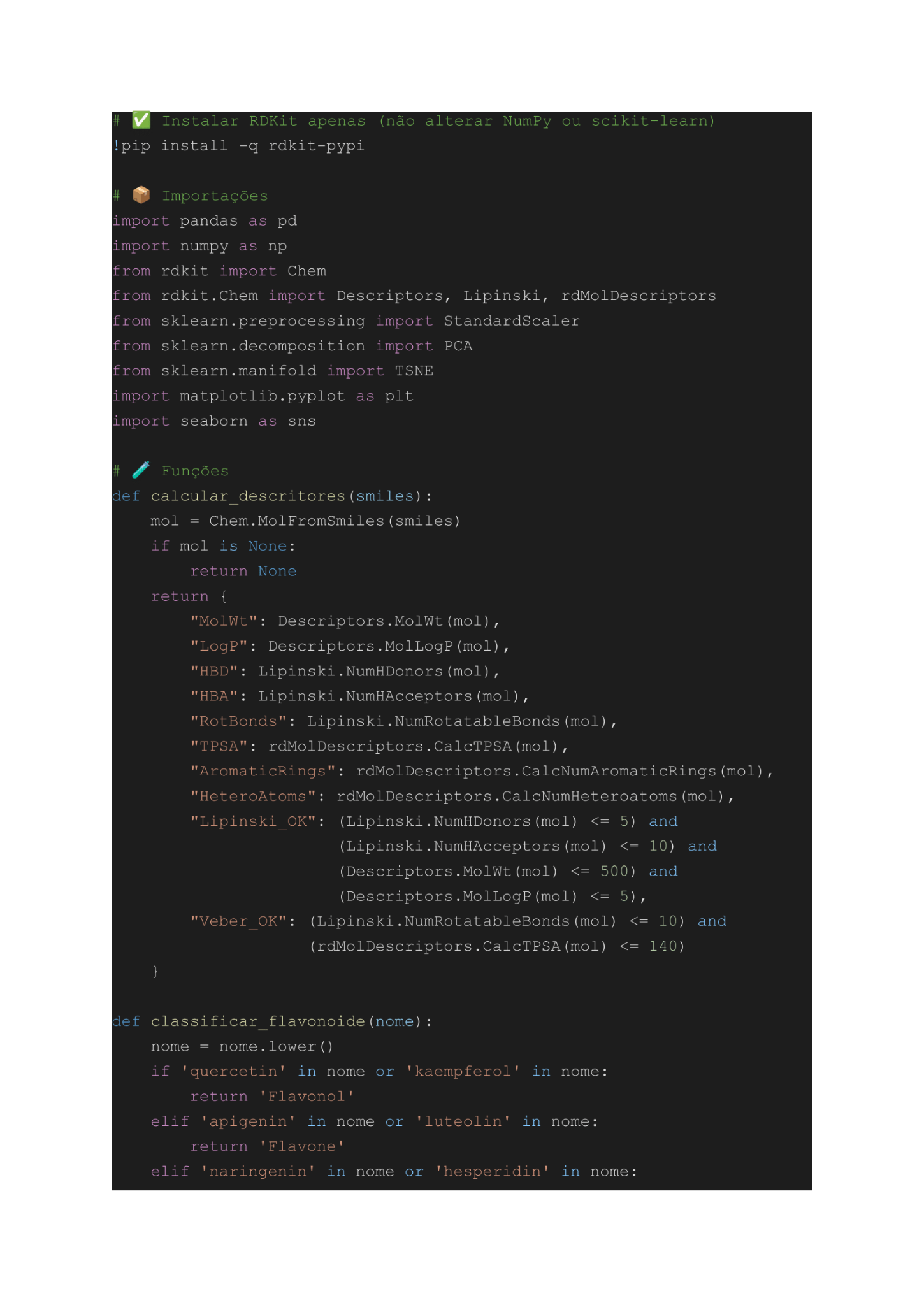


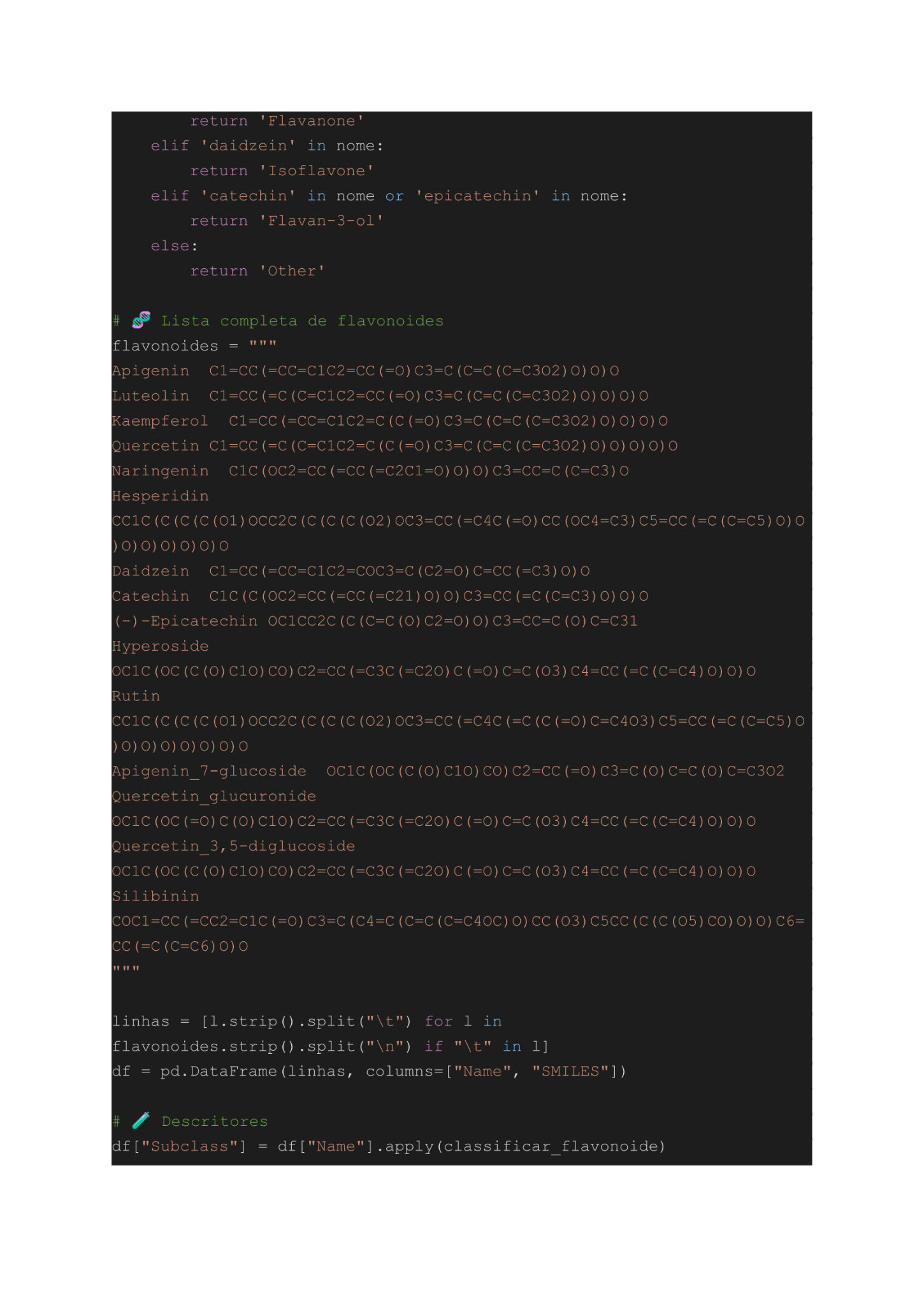

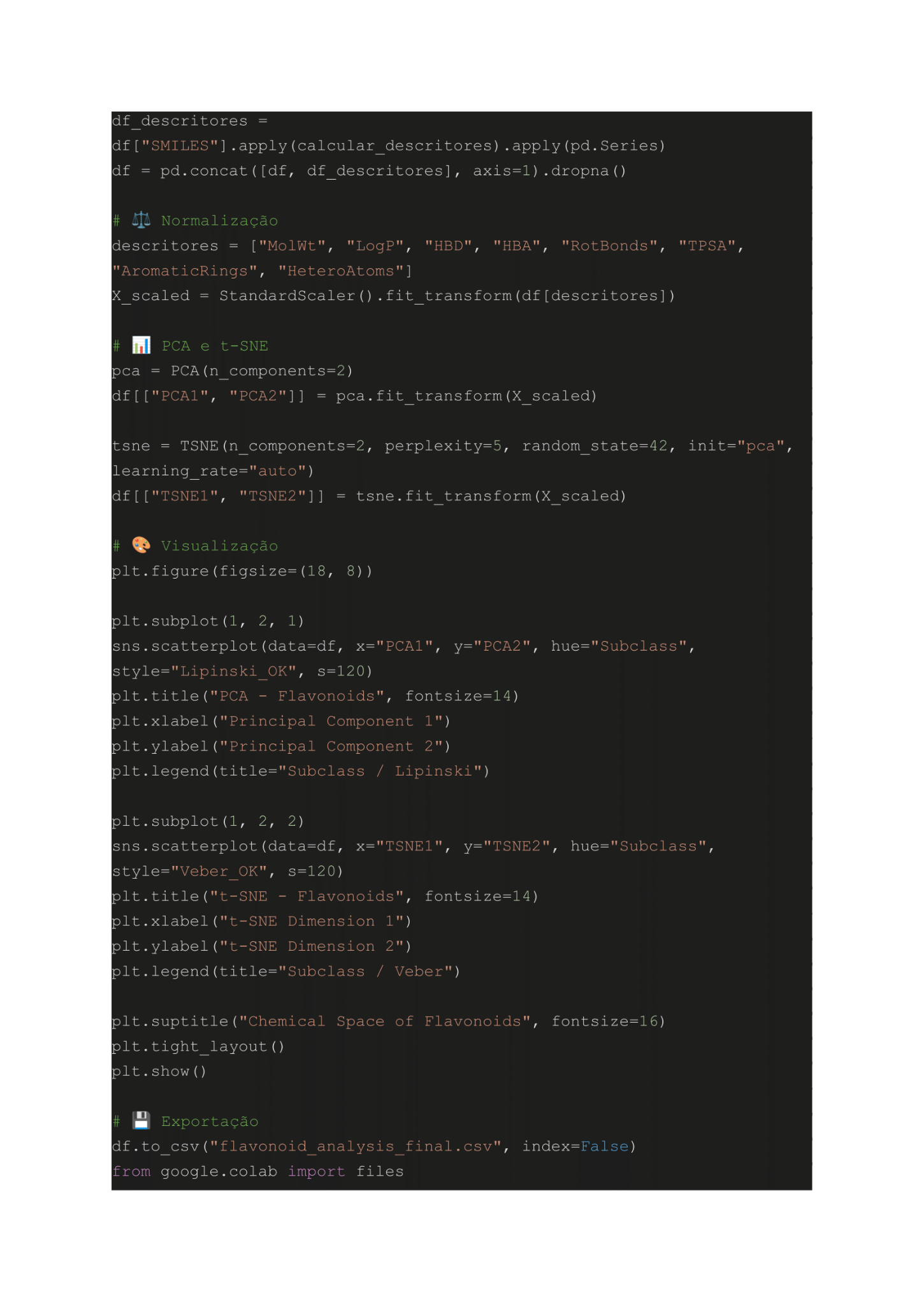


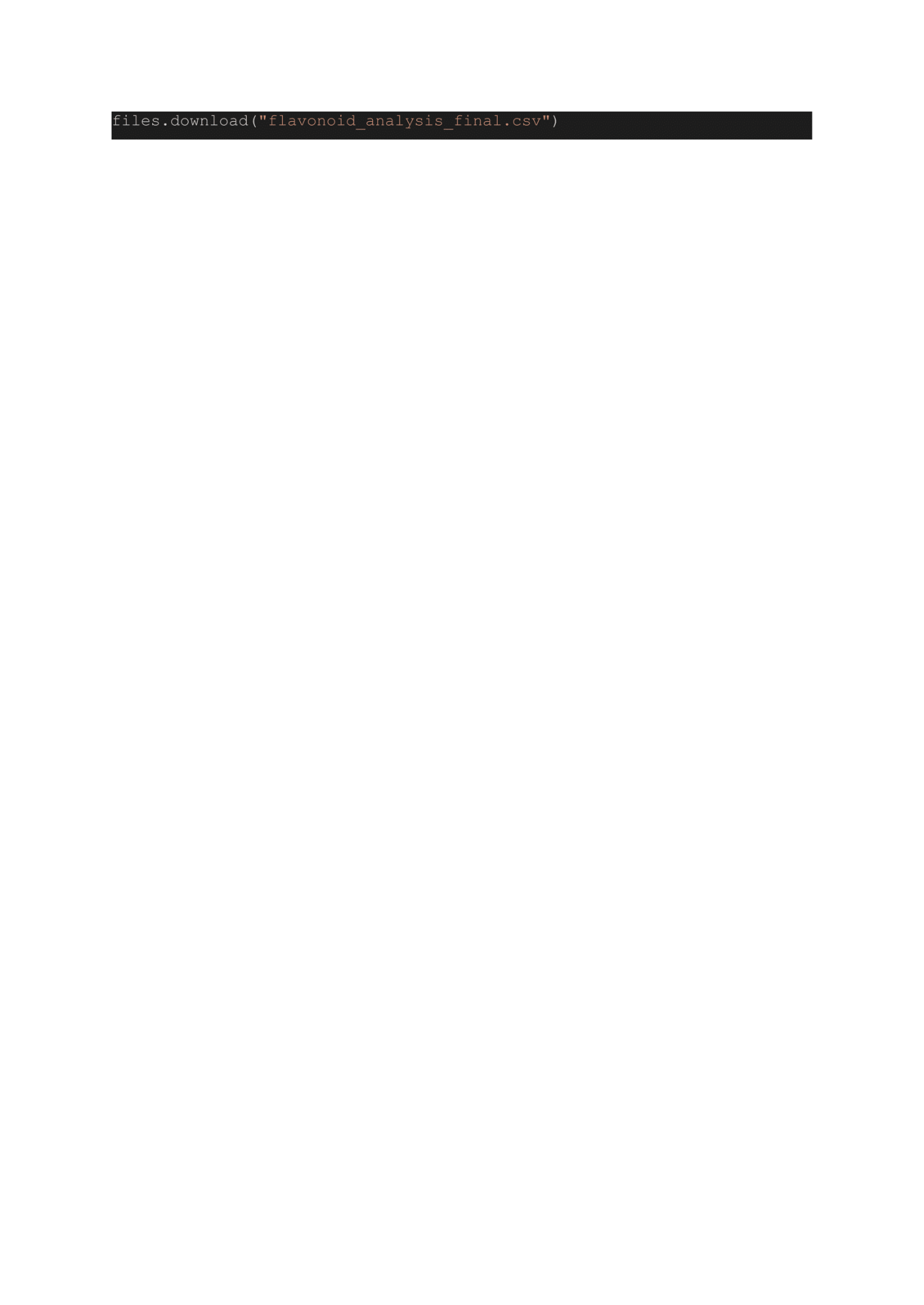

Supplement: Supplementary file 1 — Supporting File 1: Table S1: Authors, type of extract, extraction method, phytochemical analysis and analysis method. Figure S1: Python codigs used for Chemical space analysis. [file CBDV-23-e02471-s001.docx]
